# Genome-Scale Identification of Essential Metabolic Processes for Targeting the *Plasmodium* Liver Stage

**DOI:** 10.1016/j.cell.2019.10.030

**Published:** 2019-11-14

**Authors:** Rebecca R. Stanway, Ellen Bushell, Anush Chiappino-Pepe, Magali Roques, Theo Sanderson, Blandine Franke-Fayard, Reto Caldelari, Murielle Golomingi, Mary Nyonda, Vikash Pandey, Frank Schwach, Séverine Chevalley, Jai Ramesar, Tom Metcalf, Colin Herd, Paul-Christian Burda, Julian C. Rayner, Dominique Soldati-Favre, Chris J. Janse, Vassily Hatzimanikatis, Oliver Billker, Volker T. Heussler

**Affiliations:** 1 Institute of Cell Biology, University of Bern, Bern 3012, Switzerland; 2 Wellcome Sanger Institute, Wellcome Genome Campus, Hinxton, Cambridge CB10 1SA, UK; 3 Molecular Infection Medicine Sweden (MIMS), Department of Molecular Biology, Umeå University, Umeå 901 87, Sweden; 4 Laboratory of Computational Systems Biotechnology, École Polytechnique Fédérale de Lausanne, EPFL, Lausanne 1015, Switzerland; 5 Leiden Malaria Research Group, Parasitology, Center of Infectious Diseases, Leiden University Medical Center (LUMC), Leiden 2333ZA, the Netherlands; 6 Department of Microbiology & Molecular Medicine, Faculty of Medicine, University of Geneva, Geneva 1211, Switzerland; 7 Bernhard Nocht Institute for Tropical Medicine, Hamburg 20359, Germany; 8 Cambridge Institute for Medical Research, University of Cambridge, Hills Road, Cambridge CB2, 0XY, UK

**Keywords:** malaria, *Plasmodium berghei*, metabolic network, fatty acid biosynthesis, fatty acid elongation, amino sugar biosynthesis, metabolic model, Plasmodium liver stage, genome-scale knockout screen, genome-scale metabolic model

## Abstract

*Plasmodium* gene functions in mosquito and liver stages remain poorly characterized due to limitations in the throughput of phenotyping at these stages. To fill this gap, we followed more than 1,300 barcoded *P. berghei* mutants through the life cycle. We discover 461 genes required for efficient parasite transmission to mosquitoes through the liver stage and back into the bloodstream of mice. We analyze the screen in the context of genomic, transcriptomic, and metabolomic data by building a thermodynamic model of *P. berghei* liver-stage metabolism, which shows a major reprogramming of parasite metabolism to achieve rapid growth in the liver. We identify seven metabolic subsystems that become essential at the liver stages compared with asexual blood stages: type II fatty acid synthesis and elongation (FAE), tricarboxylic acid, amino sugar, heme, lipoate, and shikimate metabolism. Selected predictions from the model are individually validated in single mutants to provide future targets for drug development.

## Highlights

• 1,342 barcoded *P. berghei* knockout (KO) mutants analyzed for stage-specific phenotypes
• Life-stage-specific metabolic models reveal reprogramming of cellular function
• High agreement between blood/liver stage metabolic models and genetic screening data
• Essential metabolic pathways for parasite development and mechanistic origin revealed

## Graphical Abstract

**Figure:**
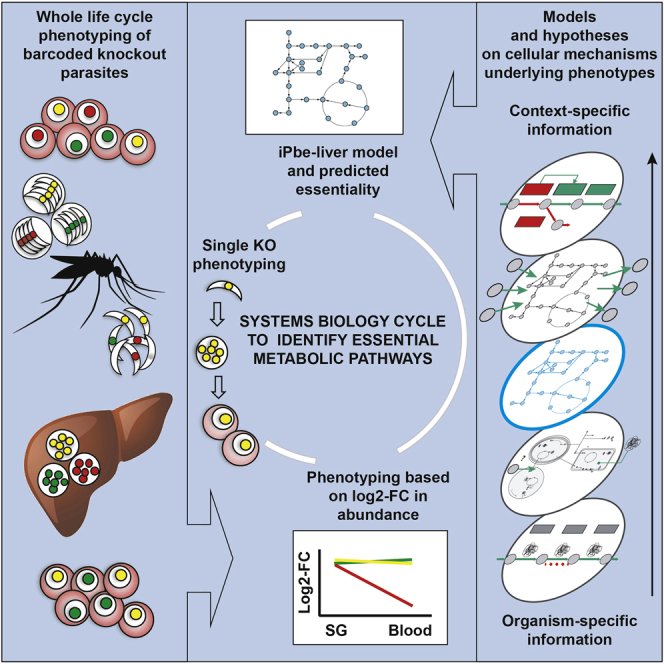

## Teaser

A genome-scale knockout screen identifies genes important for the entire malaria parasite life cycle and reveals growth bottlenecks in *Plasmodium* to establish infection in mammalian hosts.

## Introduction

Malaria, caused by parasites of the genus *Plasmodium*, remains a disease of major significance to global public health. Despite increased attention and funding, malaria still kills about half a million people each year, and the combination of drug and insecticide resistance slows down progress against this deadly disease (World Health Organization, 2018). Infection with *Plasmodium* parasites occurs through the bite of infected *Anopheles* mosquitoes, which inject motile sporozoites when feeding on blood. A proportion of them reaches and successfully invades hepatocytes. Over the course of two to five days, depending on the *Plasmodium* species, the parasite increases dramatically in size and eventually gives rise to thousands of daughter merozoites. With this immense and rapid expansion, parasites need to be highly metabolically active, despite their dependence on the host cell for nutrient acquisition. The merozoites are released into the bloodstream, where they invade red blood cells and undergo repeated rounds of asexual replication, each round culminating in the release of further invasive merozoites. It is the blood phase of the parasite life cycle that leads to the symptoms of malaria and, in the case of *Plasmodium falciparum*, can cause fatal disease (reviewed in Cowman et al., 2016). Rather than undergo asexual replication, some merozoites will instead differentiate into sexual stages of the parasite, the male and female gametocytes. Upon uptake by susceptible mosquitoes, these gametocytes are activated to form gametes and following fertilization and escape from the mosquito midgut, the parasite encysts between the epithelial midgut wall and the basal lamina. Within the oocyst, thousands of motile sporozoites are produced over the course of 7 to 10 days in a process known as sporogony. Motile sporozoites are liberated into the haemocoel of the mosquito and eventually accumulate in the salivary glands, where they await injection into a new mammalian host.

For many years, the primary focus of malaria research has been the pathogenic blood stages, and all but two of the commercially available antimalarial drugs primarily target blood-stage infection. While this has been an effective strategy, *P. falciparum* has repeatedly and rapidly developed resistance to all available blood-stage drugs, including the current frontline antimalarial, artemisinin (Blasco et al., 2017). New drugs are urgently required. Targeting the pre-erythrocytic stage of the parasite has the considerable advantage that successful drug treatment would prevent any clinical disease symptoms and could also be used to clear dormant liver stages of the *Plasmodium vivax* parasite, which can re-activate to establish blood-stage infection many years after the original mosquito bite (Campo et al., 2015). A recent screen has begun to identify dozens of candidate compounds that target the liver stage, some with great specificity (Antonova-Koch et al., 2018). However, difficult experimental models and the limited nature of our understanding of liver stage metabolism now pose major challenges for identifying their modes of action. The high metabolic activity that enables parasites to expand rapidly from a single sporozoite to tens of thousands of daughter merozoites presents a major vulnerability. Metabolic differences between pre-erythrocytic *Plasmodium* and their human host cells are known to exist (Shears et al., 2015) and could in theory be exploited for drug development, but there are currently significant gaps in our understanding of liver-stage metabolism.

Identifying genes with key roles in liver stage development that are potential drug targets requires the scaling up of experimental genetics and subsequent phenotyping during this poorly accessible stage. In *P. berghei*, a resource of >2,900 individually barcoded gene knockout vectors is now available (https://plasmogem.sanger.ac.uk/). These vectors integrate efficiently into the genome due to their long homology arms (Pfander et al., 2011) and in our experience are not maintained episomally, such that detection of a barcode after drug selection is highly indicative of the presence of a specific knockout mutant in the selected parasite populations (Gomes et al., 2015). Using barcode counting on a next-generation sequencer (barseq), we previously determined growth-rate phenotypes for the generated knockout mutants specifically during the asexual blood stages, identifying >1,360 non-essential genes from more than 2,500 screened genes (Bushell et al., 2017).

In this study, we generated pools of these blood-stage-viable knockout mutants and analyzed their phenotypes throughout the entire parasite life cycle for the first time. Using barcode sequencing, we measured changes in the relative abundance of knockout mutants in midgut oocysts, salivary gland sporozoites, and in mice following injection of sporozoites, revealing stage-specific functions for 461 genes, including transcription factors, structural proteins, and enzymes. We combined the data of the genetic screen with a liver-stage transcriptome (Caldelari et al., 2019) to generate a liver-stage metabolic model for *P. berghei* (iPbe-liver). We used this model to examine the reasons underlying the observed loss-of-function phenotypes and provide new insights into liver-stage physiology, systematically predicting thermodynamic bottlenecks, genetic interactions, and growth-limiting nutrients. To validate hypotheses generated from this model, we produced and analyzed individual knockout mutants for 20 genes and compared their phenotypes with their model-predicted essentiality.

## Results

### Validating Barseq for Analysis of Gene Knockout Mutants in Non-erythrocytic Stages

**Figure 1:**
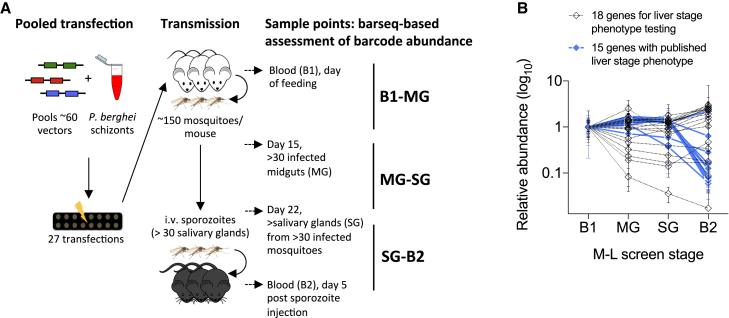
Barseq Identifies Pre-erythrocytic Phenotypes (A) Schematic showing the gene knockout (KO) screen to identify mosquito-liver stage (M-L) phenotypes. *P. berghei* schizonts were transfected with pools of barcoded *Plasmo*GEM knockout vectors and parasites selected by drug-treatment in mice. Infected mice, from which blood was sampled for barcode sequencing (B1), were used to infect mosquitoes. Midguts (MG) and salivary glands (SG) of infected mosquitoes were then sampled, and salivary gland sporozoites were collected from mosquitoes to infect mice by i.v. injection. Blood from sporozoite-infected mice (B2) was also collected for barcode sequencing. Barcode counts determined by sequencing PCR amplicons were used to determine the relative abundance of each gene knockout parasite at the life-cycle transitions shown. (B) Abundance of gene KOs at different life-cycle stages in a pilot screen, shown relative to their initial abundance at B1. Genes included in the pilot screen are shown in Table S1. Error bars represent standard deviations.

Only the asexual blood stages of *Plasmodium* parasites can be propagated continuously to drug-select for knockout mutants, meaning that only genes that are not required for blood-stage development can be investigated at later stages of the cycle using barseq. Extending barseq phenotyping beyond blood stages faces three potential obstacles: (1) population bottlenecks, (2) changes in ploidy following gamete fusion in the midgut, and (3) segregation of mutant alleles in the oocyst. In a pilot screen, we first tested whether barcoded alleles could be transmitted robustly through the population bottleneck posed by the only approximately 400 oocysts that in our hands form on average on each infected *Anopheles stephensi* midgut. Using knockout vectors targeting 15 genes with known functions at the liver stage and 19 control and test genes (shown in Table S1), a pool of mutant parasites was generated by transfection and used to infect three mice. Blood samples from each mouse were collected to establish the starting composition of mutants after drug selection (sample B1). 120–150 female mosquitoes were then allowed to feed on each mouse, and midguts (MG) from >30 mosquitoes were dissected 15 days post-infection, followed by salivary gland (SG) collection at day 22 post-infection from at least 60 mosquitoes. Half of these SGs were used to prepare a barseq library to establish the composition of the mutant pool in SG; the other half were used to collect sporozoites to infect another mouse. From this mouse, a blood sample (B2) was taken 5 days after intravenous injection of sporozoites to establish the composition of the mutant pool in B2, allowing assessment of parasite development in the liver (Figure 1A).

The relative abundance of gene knockouts in the pilot dataset (Table S1) showed at least a 5-fold drop in relative abundance between SG and B2 specifically for genes known to have a critical role at the liver stage (more than 10-fold for PALM, UIS4, aLipDH, B9, P36, P36P, FabB/F, FabZ, and TRAP; more than 5-fold drop for SLARP, PLP1, and LISP1). An 11-fold drop in relative abundance was additionally seen for one of the individually selected genes in this pilot experiment, LipA, a gene not previously studied at the liver stage, revealing for the first time a potential liver-stage role for this enzyme (Figure 1B; Table S1).

Having recapitulated published liver-stage phenotypes, we expanded the screen to cover all *Plasmo*GEM-targetable genes that are not essential at the asexual blood stage in what is hereafter referred to as the mosquito-stage liver-stage (M-L) screen. To minimize random losses of barcodes through non-representative sampling, the pool size was limited to 60 mutants, and each pool was studied in three independent transmission experiments (Figure 1A). In the absence of suitable control genes known to lack knockout phenotypes at all developmental stages, we normalized the stage-specific conversion efficiency of each mutant to the quartile of most effectively converting mutants in each set. We additionally corrected SG-B2 conversion rates using the known blood-stage growth rate of each mutant (Bushell et al., 2017) to detect pre-erythrocytic phenotypes more specifically. In total, the screen involved 1,379 vectors, transfected in 27 pools (Table S2). It required dissection of >7,000 mosquitoes and barseq of more than 600 PCR amplicons.

### Screening in Pools Rescues Known Mosquito Stage Phenotypes and Reveals Later Defects in Pre-erythrocytic Development

**Figure 2:**
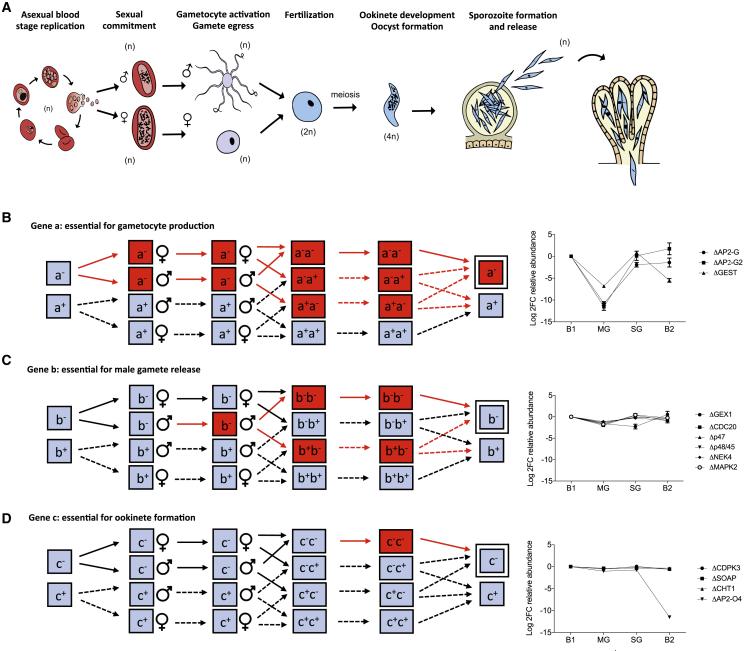
Impact of Sexual Reproduction and Ploidy on the Transmission of KO Alleles (A) Schematic illustrating ploidy changes during sexual and mosquito stages (adapted with permission from Lee et al., 2014). (B) Illustration of inheritance where KO of gene a leads to a strong reduction in fertility in both sexes. Reduced transmission (red) of a^-^ from less fertile gametes is not rescued (dotted arrows) by cross-fertilization with a^+^ parasites (solid arrows), leading to much reduced inheritance of the a^-^ allele. The line graph displays screen data from known fertility genes showing strong reductions of the corresponding barcode (strongly negative log_2_FC) among midgut (MG) oocysts. (C) As in (B), but assuming a sex specific fertility phenotype for gene b, allowing the b^-^ alleles to be transmitted effectively by the fertile sex. The line graph shows real data for genes with known functions, illustrating how the expected log_2_FC or −1 is barely noticeable. (D) Similar illustration for a hypothetical gene c with known function in ookinete or oocyst development. Inheritance of c^-^ allele may be almost unhindered due to heterozygous rescue. Real data are plotted for genes whose homozygous disruption is known to block ookinete development or infectivity. Error bars in the line graphs shown in (B), (C), and (D) show standard deviations from three replicate transmissions of the same mutant pool.

To interpret the data from a transmission screen, we considered how changes in ploidy following gamete fusion in the midgut and segregation of mutant alleles in the oocyst (Figure 2A) would affect how knockout alleles are transmitted. Mutants of the transcriptional regulators AP2-G and AP2-G2, are known to lack fertile gametocytes of both sexes (Sinha et al., 2014) and in the screen were therefore only poorly transmitted to oocysts (Figure 2B). The same was true for GEST, the gametocyte egress and sporozoite traversal gene (Talman et al., 2011), which showed both a B1-MG and a SG-B2 phenotype, consistent with its published functions (Figure 2B). In contrast, cross-fertilization between different mutants in the bloodmeal limited the power of the screen to reveal gene functions during the subsequent diploid and polyploid stages (i.e., zygotes, ookinetes, and oocysts). For instance, knockout mutants in which only one sex is sterile (Ning et al., 2013, Bennink et al., 2016) can transmit their barcodes to the oocyst by inheritance through the fertile sex (Figure 2C). As a result, reductions in barcode abundance for these sex-specific knockout mutants often did not reach significance at the B1-MG conversion.

Known gene functions in the polyploid ookinete were also generally not recapitulated in the screen, presumably due to heterozygous rescue (Figure 2D). While these observations highlight the need for future screens to be designed specifically to reveal sexual and mosquito-stage phenotypes, they also rationalize how knockout alleles of genes functioning in fertility or ookinete and oocyst development can be transmitted to salivary gland sporozoites to reveal additional gene functions after sporozoite transmission to the vertebrate host. This is illustrated by AP2-O4, a putative transcriptional regulator of oocyst maturation (Modrzynska et al., 2017) whose phenotype is rescued in the polyploid oocyst until the SG stage, but then, the haploid knockout sporozoites show an ∼3,000-fold loss during transmission back to mice, revealing a new function for AP2-O4, possibly at the liver stage (Figure 2D; Table S2).

Since in *Plasmodium* all products of meiosis are propagated into the oocyst, which remains functionally heterozygous until alleles segregate at the point of sporogony, it is likely that sporozoites lacking an essential gene can inherit sufficient protein from the oocyst to survive. TRAP (thrombospondin-related adhesive protein), which is required for sporozoite gliding, entry into salivary glands and hepatocyte invasion (Sultan et al, 1997) might be an example of protein inheritance from heterozygous oocysts to sporozoites. An ∼4-fold reduction in SG sporozoites in our screen (Figure 1B; Table S1) contrasts with a 34-fold reduction in sporozoite numbers of the TRAP gene knockout clone in the previous study, possibly because TRAP protein obtained by the sporozoite from heterozygous oocysts alleviates the phenotype of the knockout.

**Figure 3:**
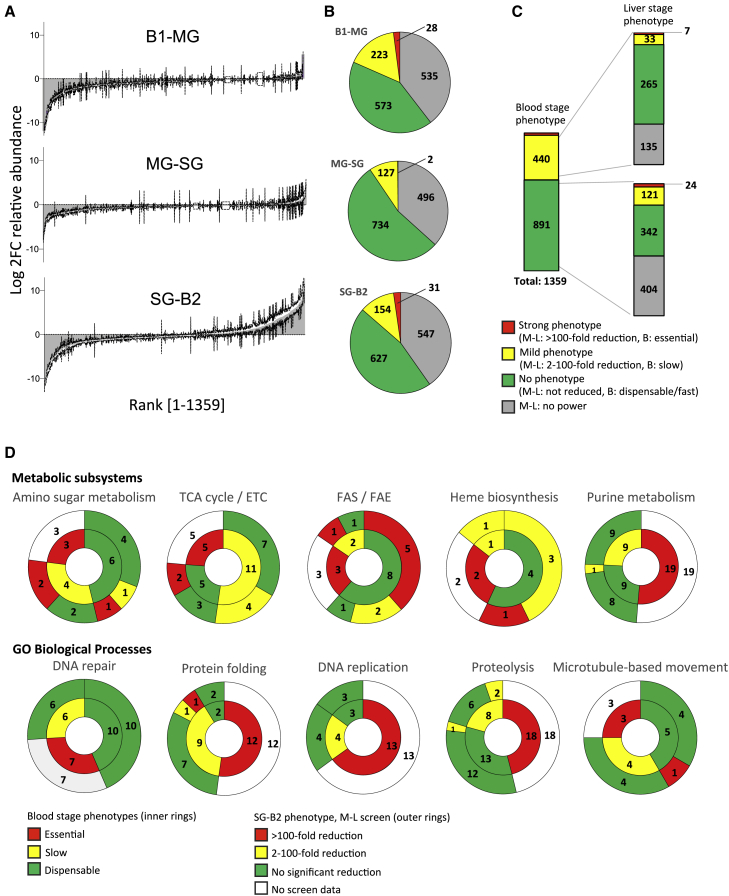
M-L Screen Gene Knockout Phenotypes at the Liver Stage Show a Bias toward Genes with Predicted Metabolic Function (A) Ranked, normalized log_2_FC values at each stage transition and for all genes with data. SG-B2 data were corrected for blood stage fitness, and an apparent increase in some mutants (right end of distribution) results from some overcompensation for slow growth. Error bars show standard deviations. (B) Pie charts show the distribution of phenotypes (see C for legend). (C) SG-B2 phenotypes shown separately for genes that at the blood stage (Bushell et al., 2017) grow slowly (yellow) or are indistinguishable from wild type (green). (D) Plots showing the blood-stage (inner ring) and liver-stage (SG-B2, outer ring) phenotypes for genes pertaining to specific metabolic subsystems (upper row) or GO biological processes (lower row). Liver-stage phenotypes for genes are clustered according to their corresponding blood-stage phenotype. The association of genes to metabolic subsystems is based on iPbe. GO biological process data, where available, are displayed in Table S2.

The same phenomenon is unlikely to extend to all sporozoite expressed genes, because once inside the salivary glands, sporozoites reprogram transcription from their now once more haploid genome in preparation for transmission back to the vertebrate host (Mikolajczak et al., 2008). At this phase of the life cycle, the ability of the screen to reveal phenotypes was therefore predicted to increase, which is confirmed by a comparison of ranked effect sizes, which are much greater for the SG-B2 transition as compared to the MG-SG conversion (Figure 3A). By first approximation, we will assume losses of mutants at the SG-B2 transition to reflect gene functions at the liver stage in the broadest sense, i.e., starting with sporozoite transmigration and invasion of hepatocytes and culminating in the release of merozoites into the bloodstream. A more precise elucidation of gene functions will require analysis of single knockout mutants (see below).

### Liver-Stage Phenotypes Are Enriched for Genes with Metabolic Functions

**Figure S1:**
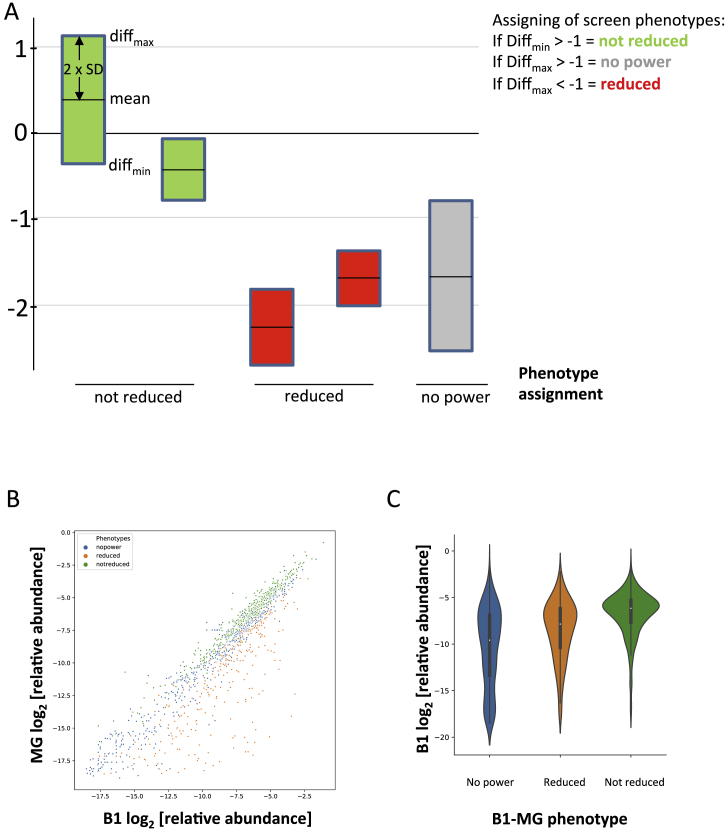
Phenotype Assignment and Statistical Analysis, Related to Figure 1 (A) A scheme showing how phenotypes of not reduced, no power or reduced were assigned to each gene knockout at each stage transition. This was based on the normalized (and blood stage fitness-corrected for SG-B2) relative change in abundance within the pool (Log2-FC) and associated standard deviation. A Log2-FC (−2XSD) value was calculated by the subtraction of 2xSD from the diff value. A Log2-FC (+2XSD) value was calculated by the addition of 2xSD to the diff value. Genes with a “not reduced” phenotype have a Log2-FC (−2XSD) value > -1. Genes with a no power phenotype have a Log2-FC (+2XSD) > -1. Genes with a reduced phenotype have a Log2-FC (+2XSD) value of < -1. The two “not reduced” bars serve to illustrate how both a small effect size or a high variance can lead to a conservative phenotype call of “not reduced” where the mean remains close to 1. While the first “reduced” bar shows a clear reduced phenotype, the second “reduced” bar and its comparison with the “no power” bar shows how at a given log_2_FC, variance can determine whether a low stage transition rate is called “reduced” or “no power.” (B) Mean relative abundance of all mutants in B1 and MG samples. A high level of correlation shows representative sampling by the mosquito for all but the least abundant mutants. Color-coding of phenotypes showing that underrepresented mutants lack statistical power to make a phenotype call. (C) Violin plots showing that phenotypes at the B1-MG transition were assigned preferentially for the well-represented mutants in the B1 sample, which were sampled accurately by the mosquito.

Taking a conservative approach to calling phenotypes that takes into account both the effect size and the variance across biological triplicates as illustrated in Figure S1, we find that at each transition, only a small proportion of mutants (9%–18%) are significantly depleted, while for the majority of genes, we can either be confident that they are “not reduced” or the statistical power is considered insufficient to make a clear call (Figure 3B). Of the 1,359 mutants for which data was obtained, 898 showed no significant reduction at any transition. At the B1-MG, MG-SG, and SG-B2 transitions, 251, 129, and 185 mutants, respectively, showed reduced stage conversion (Figure 3B). Statistically robust transmission phenotypes were revealed, regardless of whether mutants were previously found to have normal or slow growth at the asexual blood stage (Figure 3C). The latter does not, therefore, appear to be a major confounder of our ability to detect phenotypes during the rest of the life cycle.

Mutants that were reduced strongly (>100-fold) at the SG-B2 transition showed a remarkable enrichment (p < 0.01) for metabolic genes (15 of 31 genes in this category encoded enzymes versus only 4 expected; Table S2). Some of the pathways represented by lost mutants are consistent with the known importance of heme and fatty acid biosynthesis at the liver stage (Shears et al., 2015, Goldberg and Sigala, 2017); others implicate more unexpected roles for fatty acid elongation, amino sugar metabolism, and the electron transport chain (Table S2; Figure 3D). By comparison, we did not see liver-stage-specific essentiality for genes with functions in DNA repair, DNA replication, or proteolysis (Figure 3D).

### Stage-Specific Genome-Scale Metabolic Models for *P.* *berghei*

**Figure S2:**
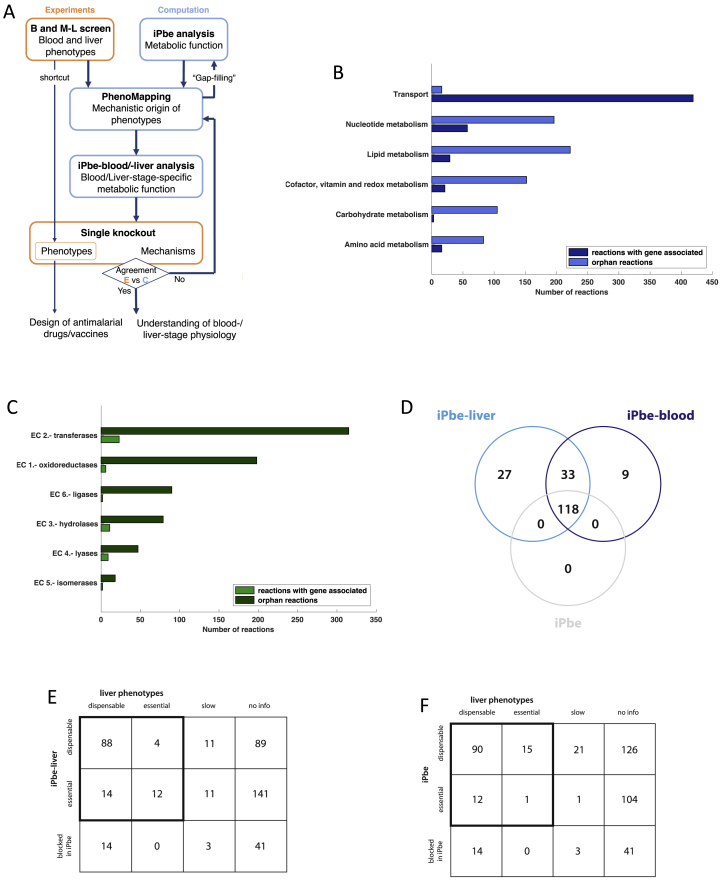
Combined Experimental and Computational Workflow to Study Blood and Liver Phenotypes and Their Mechanistic Origin using iPbe. Description of the iPbe Model and Essentiality Predictions, Related to Figure 4 (A) Workflow diagram showing how data form the experimental screening platform are integrated to study blood and liver phenotypes and their underlying mechanisms and to develop a more comprehensive metabolic model following the cycle of systems biology. (B) Distribution of metabolic enzymes in iPbe. (C) Metabolic subsystems in iPbe. (D) Relation between genes predicted as essential in iPbe, iPbe-blood and iPbe-liver. (E) Contingency matrix for gene essentiality predictions and the liver stage M-L screen phenotypes compared with iPbe liver. (F) Contingency matrix as for (E) but compared with the general iPbe model.

**Figure 4:**
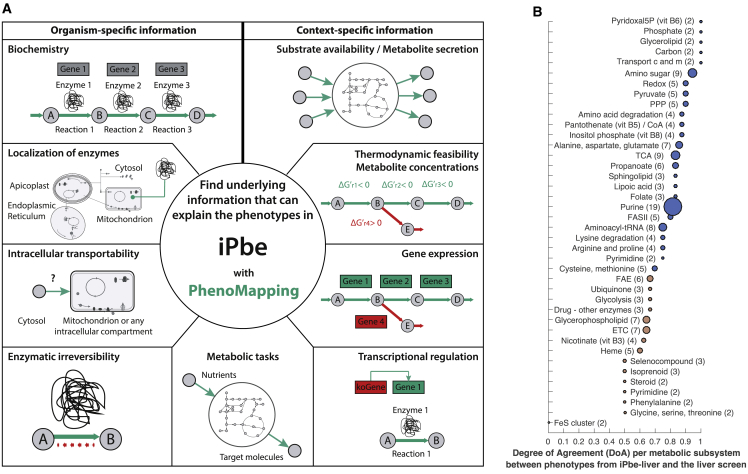
The PhenoMapping Workflow and Degree of Agreement for Metabolic Subsystems in iPbe-Liver with the M-L Screen (A) Illustration of the PhenoMapping workflow for the integration of organism- and context-specific information into the genome-scale iPbe metabolic models. Context-specific information denotes life-cycle stage-specific processes, such as gene expression, as well as environmentally specific factors, such as substrate availability. Metabolic tasks are at the interface between organism- and context-specific information. The production of molecules, such as amino acids, fatty acids, nucleotides, etc., is required for growth independent of the context, but the ratios in which they are required might change with the growing conditions or life stage. See STAR Methods and Table S4 for a detailed description of iPbe. (B) Degree of agreement (DoA) between the gene essentiality predictions in iPbe-liver and the experimental phenotypes at the SG-B2 transition. Metabolic subsystems are ranked by level of agreement. Numbers show genes with screen data per subsystem (needs to be >1 for inclusion).

With metabolism emerging as a defining feature of the SG-B2 transition, we decided to construct a genome-scale model of *P. berghei* metabolism to evaluate the screen results systematically in the context of current knowledge. As with our previous general *P. falciparum* model (iPfa) (Chiappino-Pepe et al., 2017a), we based the *in silico P. berghei* (iPbe) model on a set of metabolic tasks (Table S3) and on annotated metabolic gene functions (Table S4). We build upon this computational framework through a process we call PhenoMapping (STAR Methods). In a unique decomposition approach, we consider separately different layers of information, such as nutrient availability, gene expression, and gene knockout phenotypes in order to refine the model, for instance, by adding missing enzymatic or transport capabilities (Figure S2A). We initially used asexual blood-stage growth rates of Bushell et al. (2017) and subsequently incorporated the phenotypes from the SG-B2 transition of the current screen (STAR Methods). The iPbe model integrates 428 genes and 1,318 reactions (transport and enzymatic reactions; Figures S2B and S2C) that reflect available knowledge and new postulates on the metabolism of the parasite based on our PhenoMapping analysis. We used the iPbe model to analyze essential metabolic capabilities in a stage-specific manner (Figure 4A), working under the assumption that most metabolic phenotypes at the SG-B2 transition reflect gene functions during liver-stage development, a prediction we will validate experimentally below.

To evaluate computationally the essentiality of the 428 genes in iPbe by PhenoMapping, we initially assumed unlimited transport capabilities, but we worked with the known range of metabolite concentrations and gene expression levels (Caldelari et al., 2019, Otto et al., 2010, Teng et al., 2009, Teng et al., 2014, Vo Duy et al., 2012), and we considered the potential for dynamic regulation of gene expression between isoenzymes (Figure 4A; STAR Methods). A stage agnostic model initially predicted 155 of the 428 genes as essential in at least one condition (Table S3). To create blood- and liver-stage-specific models, we used existing knowledge of host metabolite availability as constraints to identify the combinations of nutrients the parasite would need to access to maximize agreement with the phenotypes of the respective knockout screens. We allowed iPbe to uptake 90 metabolites from the surroundings (i.e., the hepatocyte), and we integrated thermodynamic data (pH of intracellular compartments and membrane potential), as well as liver stage transcriptome data (Caldelari et al., 2019), to generate a liver-stage-specific metabolic model, iPbe-liver. Analogously, an optimized thermodynamic blood-stage model, iPbe-blood, assumes uptake of 94 metabolites from the reticulocyte and integrates blood-stage metabolomic and transcriptomic data (Otto et al., 2014, Teng et al., 2009, Teng et al., 2014, Vo Duy et al., 2012) (STAR Methods; Table S4).

To generate insights into the stage-specific metabolism from the data-optimized models, we first simulated *in silico* knockouts of all genes individually (Table S3), predicting lethality where the KO would lead to the absence of an essential metabolic building block. Of the 428 genes in iPbe, 178 are predicted as essential *in silico* for growth in iPbe-liver (Table S3). While 151 genes were essential in both stage-specific models, a subset of 27 genes becomes essential specifically at the liver stage (Figure S2D). These represent seven metabolic subsystems: fatty acid synthesis (FASII) and elongation (FAE), tricarboxylic acid (TCA), amino acid, heme, lipoate, and shikimate metabolism. To estimate the overall accuracy of essentiality predictions in iPbe-liver, we compared with the experimentally obtained phenotypes at the SG-B2 transition, which are available for 157 out of 428 genes in iPbe (Table S4; the remaining 271 genes being either blood-stage essential or not covered by the *Plasmo*GEM resource). iPbe-liver predicts essentiality with 85% accuracy and a Matthew Correlation Coefficient (MCC) of 0.51, the latter providing a more appropriate measure considering the different number of dispensable and essential SG-B2 phenotypes (116 and 16 respectively). Similarly, iPbe-blood is 84% accurate at an MCC of 0.7 (95 dispensable and 133 essential blood-stage phenotypes; Bushell et al., 2017). Accuracy and MCC for each stage-optimized model were higher than for a stage agnostic model (Figure S2 and STAR Methods), and MCC values were comparable to those obtained with well-studied model organisms *Escherichia coli* (Monk et al., 2017) and *Saccharomyces cerevisiae* (Heavner and Price, 2015). iPbe-liver predicted experimental knockout phenotypes accurately for over 70% of metabolic subsystems (degree of agreement > 0.7) including those for fatty acid, amino sugar, and folate metabolism (Figure 4B).

### All Enzymes in the FASII Pathway Are Crucial for the *P.* *berghei* Liver Stage

**Figure 5:**
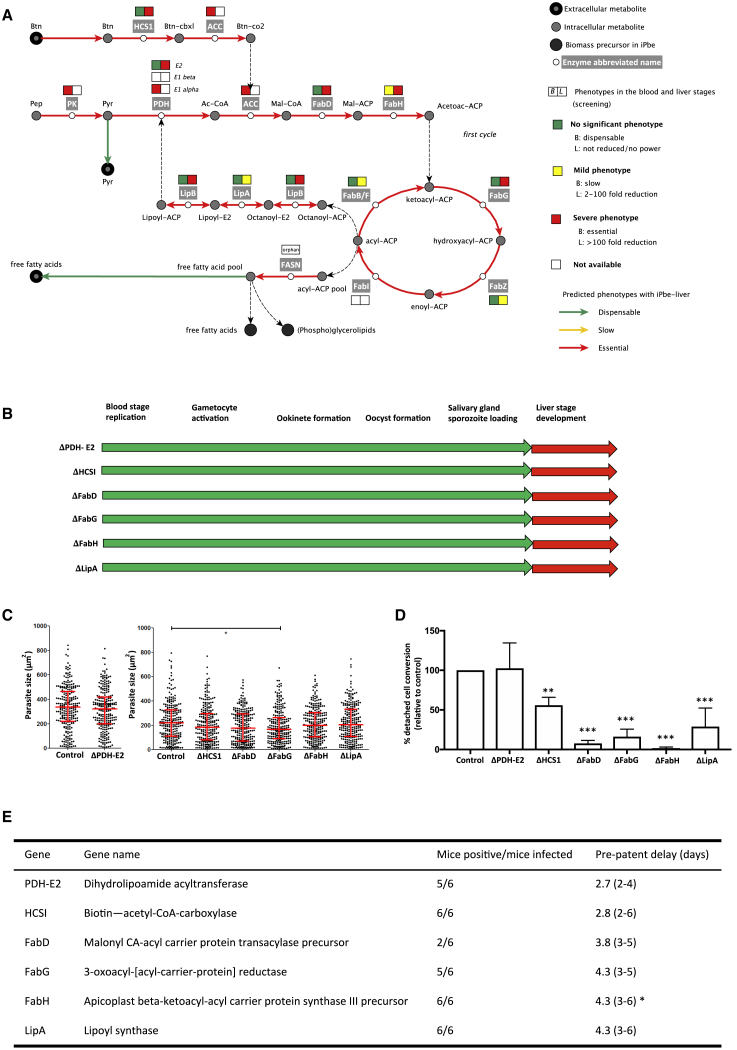
Mutations FASII, Lipoate Synthesis, and Biotin Metabolic Pathways Affect Liver-Stage Development (A) Pathway maps for FAS II, lipoate metabolism, and biotin metabolism in the *Plasmodium* apicoplast. See Table S4 for gene IDs, enzyme functions, and reactions. Pep, phosphoenolpyruvate; Pyr, pyruvate; Ac-CoA, acetyl-CoA; Mal-CoA, malonyl-CoA; Mal-ACP, malonyl-[acp]; Acetoac-ACP, acetoacetyl-[acp]; Octanoyl-ACP, octanoyl-[acp]; Octanoyl-E2, protein N6-(octanoyl)lysine; Lipoyl-E2, protein N6-(lipoyl)lysine. (B) Schematic representation of phenotypes of single knockout (KO) mutants. Green, phenotype not significantly different from wild type (WT) parasites. Red, phenotype significantly different (>2-day delay in pre-patent period). (C) Size of 250 cultured EEFs (48 hpi) per mutant; median and interquartile ranges are shown in red. ^∗^ = p < 0.05 by Kruskal-Wallis test. (D) Relative maturation of EEFs measured as conversion of infected host cells to detached cells at 48 hpi. Error bars show standard deviations from 8 biological replicates (for PDH-E2) or 3 biological replicates (all other mutants). The results were statistically evaluated by a one-way analysis of variance (ANOVA) test with Dunnet’s multiple comparisons (^∗∗^p ≤ 0.01; ^∗∗∗^p ≤ 0.001). (E) The number of mice that developed blood-stage infections after injection of 5,000 mutant sporozoites and the mean delay (range) in pre-patency compared to mice infected with WT sporozoites. ^∗^, gene KO mutants with a significantly “slow fitness” blood stage phenotype (Bushell et al., 2017). See Figure S4B for plots showing the course of blood stage infections after sporozoite injection.

**Figure S3:**
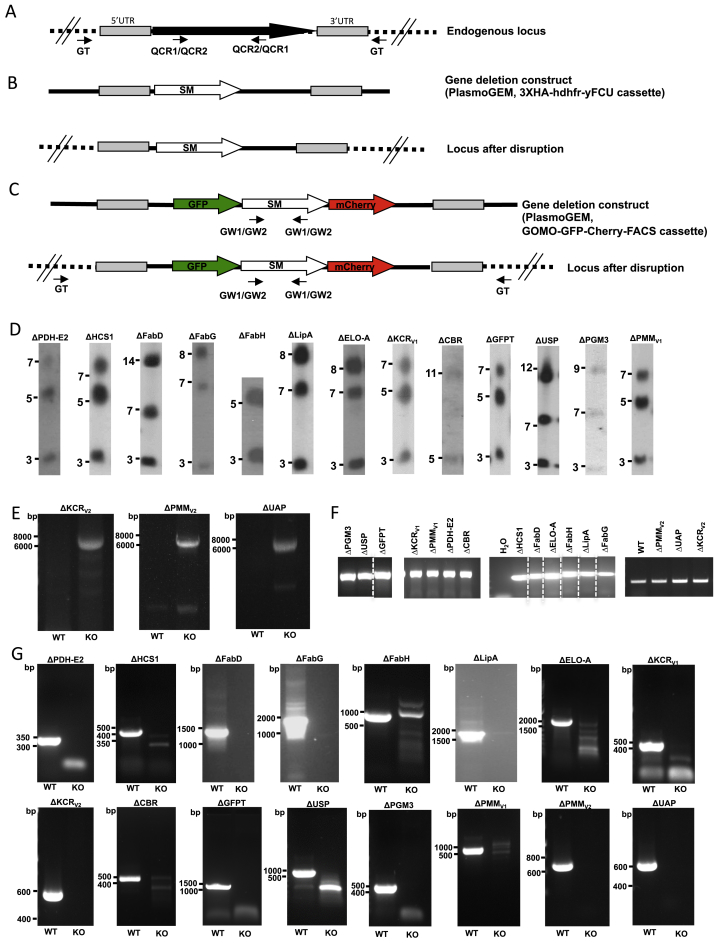
Genotyping of Single Gene Knockout Parasite Lines, Related to Figures 5, 6, and 7 (A) Schematic representation of the endogenous gene of interest (GOI) locus, (B) the gene deletion targeting construct (*Plasmo*GEM) containing the 3xHA-hdhfr-yFCU cassette and the GOI locus after disruption following double homologous recombination. Such a replacement strategy was used in cases where no sexual stage crossing was performed to bypass lethal mosquito stage phenotypes. The positions of primers used in PCRs to confirm GOI deletion is indicated by arrows labeled QCR1 or QCR2 and QCR2 or QCR1. (C) Schematic representation of the endogenous gene of interest (GOI) locus, the gene deletion construct (*Plasmo*GEM) containing the GOMO-GFP-mCherry-FACS cassette and the GOI locus after disruption following double homologous recombination. PCR primers used to confirm successful integration of the construct are indicated by arrows GT and GW1 or GW2 and PCR primers used to confirm deletion of the GOI are indicated by arrows QCR1 or QCR2 and QCR2 or QCR1 respectively. All primer sequences are shown in Table S6. The gene replacement strategy with the GOMO-GFP-mCherry-FACS cassette was used to generate mutant parasites that were crossed with a WT parasite line for cases in which the sKO was blocked at the mosquito stage. (D) Pulse field gel electrophoresis (PFGE) for the following gene knockout parasite lines (chromosome location of replaced gene shown in brackets, knockouts generated via transfection with constructs containing the 3xHA-hdhfr-yFCU cassette): ΔPDH-E2 (5), ΔHCS1 (5), ΔFabD (14), ΔFabG (8), ΔFabH (3), ΔLipA (13), ΔELO-A (8), ΔKCR_v1_ (5), ΔCBR (11), ΔGFPT (5), ΔUSP (12), ΔPGM3 (9) and ΔPMM_v1_ (5). A probe was used that recognized the 3′UTR of the pbdhfr hybridized to the knockout cassette that replaced the above genes on the chromosomes listed above. A probe of ≈800 bp fragment of the 5′UTR of the PBANKA_0508000 gene located on the chromosome 5 was also used for the ΔFabH mutant parasite and a probe of ≈800 bp fragment of the 5′UTR of the PBANKA_0508000 gene located on the chromosome 5 was additionally used for the ΔCBR mutant parasite. All images have been cropped from PFGE images showing other parasite lines. (E) Diagnostic PCR of the single gene knockout parasite lines ΔKCRv2, ΔPMMv2 and ΔUAP using primers to test for the presence of the WT locus and successful integration at the kcr, pmm and uap loci, respectively (parasites generated by transfection with a construct containing the GOMO-GFP-mCherry-FACS cassette). GT and GW2 primers were used to show integration for ΔKCRv2 and ΔPMMv2 and GT and GW1 for ΔUAP. Parasites were generated with the aim of crossing with WT parasites to rescue the lethal mosquito stage phenotype. Lanes showing markers have been removed and also other PCR products from other clones. (F) Control PCR to show successful amplification from gDNA samples taken from all single gene knockout parasite lines generated in this study Dotted lines between lanes indicate the reordering of lane images from the same gel photo. A space between lanes indicates lane images taken from separate gels. (G) PCRs showing the presence of genes in WT parasites and absence in mutant parasites for all single gene knockout lines generated in this study. All primers used for genotyping PCRs are listed in Table S6. Lanes showing markers have been removed and also other PCR products from other clones.

**Figure S4:**
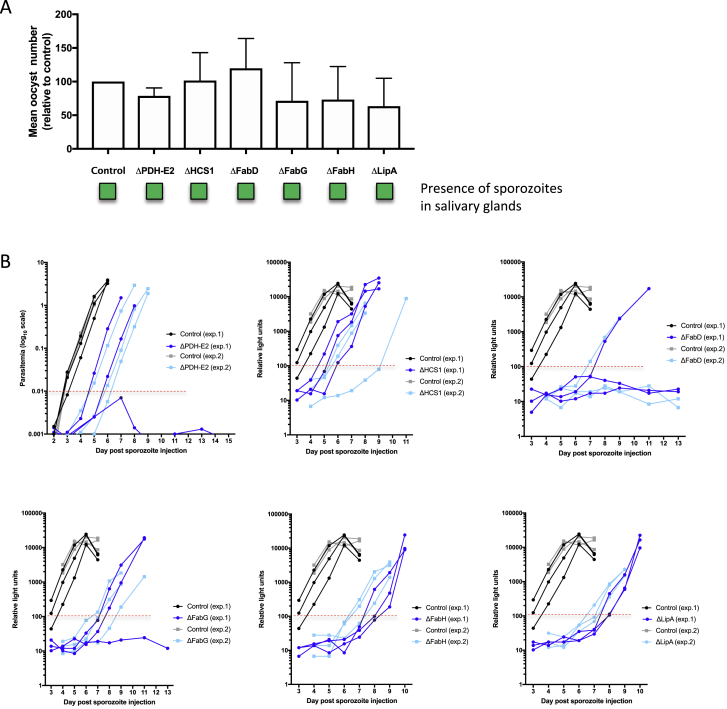
Phenotypic Analysis of Gene Knockout Mutant Parasite Lines Associated with the FASII Pathway, Related to Figure 5 (A) Graph showing oocyst numbers (relative to control; set to 100%) at day 7 post infection for ΔPDH-E2, ΔHCS1, ΔFabD, ΔFabG, ΔFabH and ΔLipA parasites. Error bars indicate standard deviation. The results were statistically evaluated by a one-way analysis of variance (ANOVA) test with Dunnet’s multiple comparisons (all results non-significant at 95% confidence). Boxes shown below the graph indicate the presence (green) of sporozoites from the salivary glands for each knockout strain between days 18 and 21 post-infection. (B) Graphs to show parasitemia for mice injected with ΔPDH-E2 sporozoites (based on FACS) or relative blood stage parasitemia for mice injected with ΔHCS1, ΔFabD, ΔFabG, ΔFabH and ΔLipA parasites (based on relative light units by luciferase assay). Data from all mice (control and KOs) are shown for two independent experiments and lines are drawn for the relative light unit level or parasitemia considered as being the point at which a mouse has become positive.

**Figure S5:**
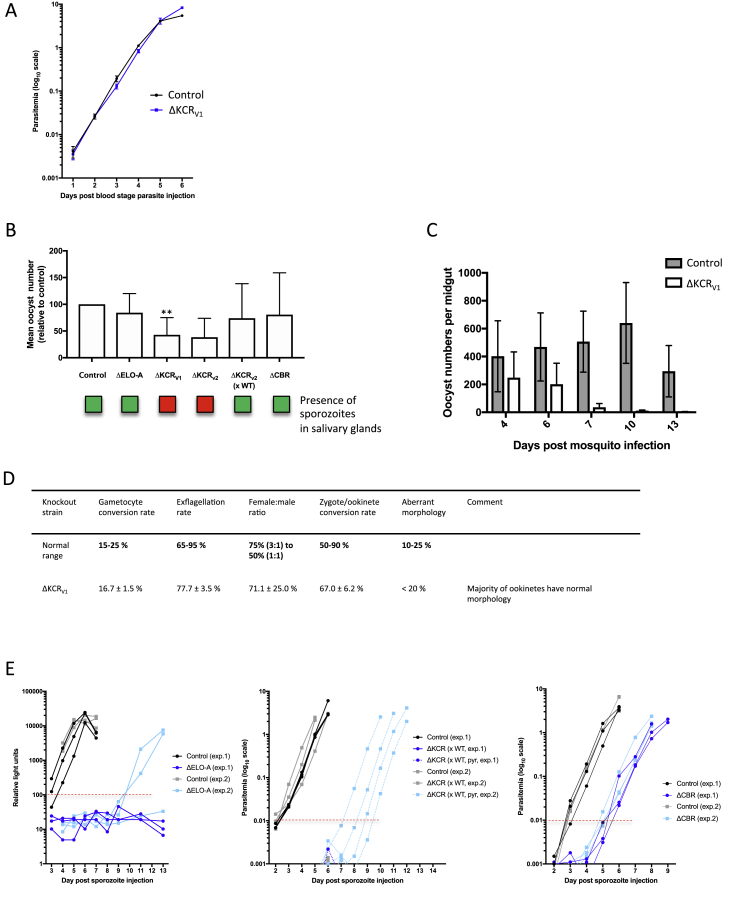
Phenotypic Analysis of Gene Knockout Mutant Parasite Lines Associated with the FAE Pathway, Related to Figure 6 (A) Graph showing growth rate of ΔKCRv1 blood stage parasites in relation to control parasites, as shown as progression of parasitemia at successive days after injection of blood stage parasites. (B) Graph showing oocyst numbers (relative to control; set to 100%) at day 7 post infection for ΔELO-A, ΔKCRv1, ΔKCRv2, ΔKCRv2 (xWT) and ΔCBR parasites. Error bars indicate standard deviation. The results were statistically evaluated by a one-way analysis of variance (ANOVA) test with Dunnet’s multiple comparisons (^∗∗^p < 0.01). Boxes shown below the graph indicate the presence (green) or absence (red) of sporozoites from the salivary glands for each knockout strain between days 18 and 21 post-infection. (C) Graph showing oocyst numbers in the ΔKCRv1 parasite strain relative to the control on days 4, 6, 8, 10 and 13 post-infection. Error bars display standard deviation. (D) Table displaying sexual and mosquito stage phenotypic data for ΔKCRv1 parasites; gametocyte conversion rate, exflagellation rate, female: male ratio, zygote to ookinete conversion rate and percentage of ookinetes showing abnormal morphology. Data is shown in relation to normal ranges for such phenotypic assessments. (E) Graphs showing relative blood stage parasitemia for mice injected with ΔELO-A parasites (relative light units by luciferase assay) and parasitemia for mice injected with ΔKCRv2 (x WT) and ΔCBR parasites (based on FACS). Data from all mice (control and KOs) are shown for two independent experiments and lines are drawn for the relative light unit level or parasitemia considered as being the point at which a mouse has become positive.

Malaria parasites harbor a type II apicoplast-localized fatty acid biosynthesis (Fab) pathway (FASII, Figure 5A; Table S4) and are also able to scavenge fatty acids from the host (Mi-Ichi et al., 2006). The lack of gene homologs in human cells had flagged the FASII pathway as a potential target for antimalarial drugs, but it was found to be non-essential for erythrocytic asexual replication (Bushell et al., 2017, Shears et al., 2015). The marked loss of FASII mutants at the SG-B2 transition (Figure 3D) confirmed previously published liver-stage phenotypes of single knockout mutants ΔFabZ and ΔFabB/F (Vaughan et al., 2009, Yu et al., 2008), but it also showed for the first time that the genes coding for FabD, FabG, and FabH are equally important with log_2_-fold reductions of −11.2, −9.2, and −9.6, respectively (Figure 5A; Table S5). Our screen predicts that genes of the E2 complex (log2-fold reduction of −9.0) and LipA also have liver-stage phenotypes (log_2_-fold reduction of −5.2; Figure 5A; Table S5). To verify barseq data, single knockout parasite lines were generated for PDH-E2, HCS1, FabD, FabG, FabH, and LipA (Figures S3 and 5B; Table S6). All mutants showed normal numbers of oocysts and produced salivary gland sporozoites (Figure S4A). Only FabG knockout mutants showed a significant difference in the size of *in vitro* exo-erythrocytic parasites at 48 h post-infection (hpi; Figure 5C). Aside from the line ΔPDH-E2, all mutants showed defects later in liver-stage development. ΔHCS1 parasites showed a 50% reduction in the formation of detached cells, a measure of the completion of *in vitro* liver-stage development, while ΔFabD, ΔFabG, ΔFabH, and ΔLipA mutants all showed even more pronounced phenotypes in detached cell formation (Figure 5D). The differences in the success of *in vitro* development were reflected by differences in phenotypes seen *in vivo*. While all mutants showed a clear delay in the pre-patent period following injection of sporozoites, this delay was stronger for ΔFabD, ΔFabG, ΔFabH, and ΔLipA mutants than for ΔPDH-E2 and ΔHCS1 mutants (Figures 5E and S4B). The *in vitro* and *in vivo* liver-stage phenotypes obtained for the FASII pathway single knockout mutants confirm the findings of the screen and the function of FASII in iPbe-liver, and they serve as a further validation that the screen is successful in revealing genes with liver-stage importance.

PhenoMapping with iPbe suggests that the knockout of FASII can lead to loss of fitness in three different physiological scenarios (Table S3). In the first, there are not enough fatty acids in the host cell to satisfy the requirements for parasite growth, and hence, the parasite must synthesize them (Figure 5A; Table S3). In the second, the parasites require a lipoylated PDH in the apicoplast and must synthesize lipoyl-ACP in this organelle through FASII. In the third scenario, FASII is required to consume excessive amounts of pyruvate produced in the apicoplast by pyruvate kinase in the process of ATP generation to fuel the protein synthesis machinery.

### Key Roles for Fatty Acid Elongation (FAE) in Mosquito and Liver Stages

**Figure 6:**
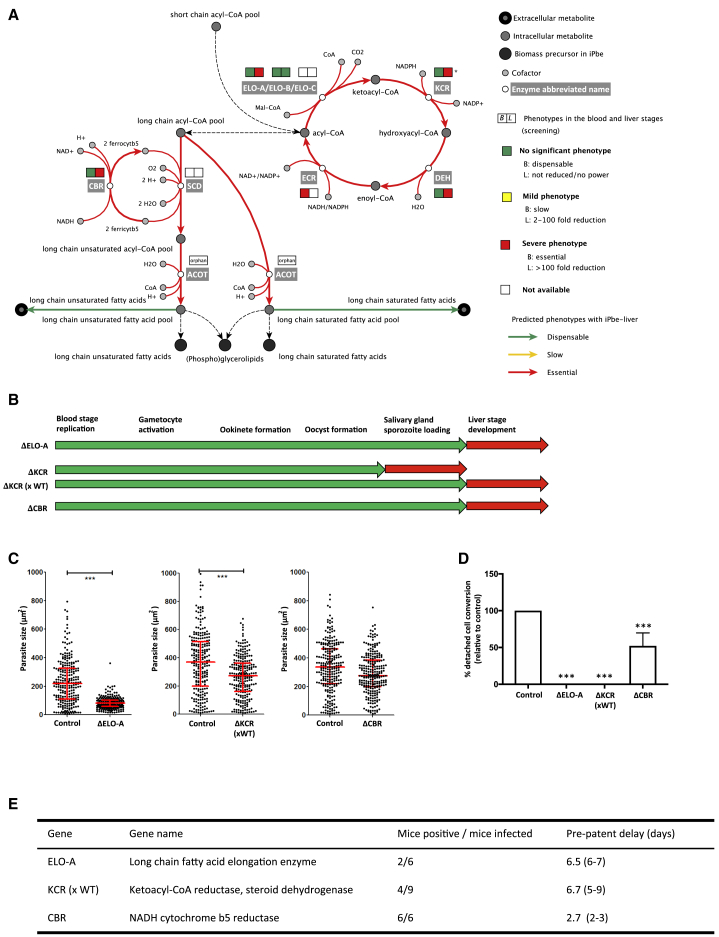
Mutations in Fatty Acid Elongation Disrupt Mosquito- and Liver-Stage Development (A) Pathway map for elongation of fatty acids (FAE) in the *Plasmodium* endoplasmic reticulum. See Table S4 for gene IDs, enzyme functions, and reactions. Blood-stage screen data suggested KCR to be essential, but we here correct the phenotype to dispensable, since a genotyped ΔKCR parasite shows comparable blood-stage growth to control parasites (Figure S5A). (B) Schematic representation of developmental blocks for single KOs and ΔKCR sporozoites derived from a ΔKCR × WT genetic cross. Green, phenotype not significantly different from WT. Red, block in life-cycle progression, except for liver stage, where red indicates phenotype significantly different from WT (>2-day delay in pre-patent period). (C) Size of cultured liver stages (48 hpi) of 250 EEFs. Median and interquartile ranges in red. ^∗^p < 0.05 by Kruskal-Wallis test. (D) Relative maturation of EEFs measured as conversion of infected host cells to detached cells at 48 hpi. Error bars show standard deviations from 3 biological replicates (for ELO-A) or 8 biological replicates (all other mutants). The results were statistically evaluated by a one-way analysis of variance (ANOVA) test with Dunnet’s multiple comparisons (^∗∗∗^p ≤ 0.001). (E) The number of mice with blood infection after injection of 5,000 sporozoites and the mean delay (range) in pre-patency compared to mice infected with WT sporozoites. ^∗^, gene KO parasites with a significantly “slow” blood-stage phenotype (Bushell et al., 2017). See Figure S5E for plots showing the course of blood-stage infections after sporozoite injection.

Short- and medium-chain fatty acids, either synthesized *de novo* or taken up from the host cell, can undergo cyclical elongation and desaturation before being integrated into phosphoglycerolipids and cellular structures. Little work has been performed on FAE in malaria parasites, which harbor a pathway in the endoplasmic reticulum (ER) that comprises three elongases (ELO-A, ELO-B and ELO-C), a ketoacyl-CoA reductase (KCR), a hydroxyacyl-CoA dehydratase (DEH), and an enoyl-CoA reductase (ECR), required for the final elongation step (Ramakrishnan et al., 2012, Ramakrishnan et al., 2013; Table S4). The gene encoding ECR has resisted deletion at the blood stage (Bushell et al., 2017), but genes encoding ELO-A, KCR, and DEH were covered in the current screen, and all are among the 11 genes with strongest reduction at the SG-B2 transition (>1,000-fold, Figure 6A; Table S5).

**Figure S6:**
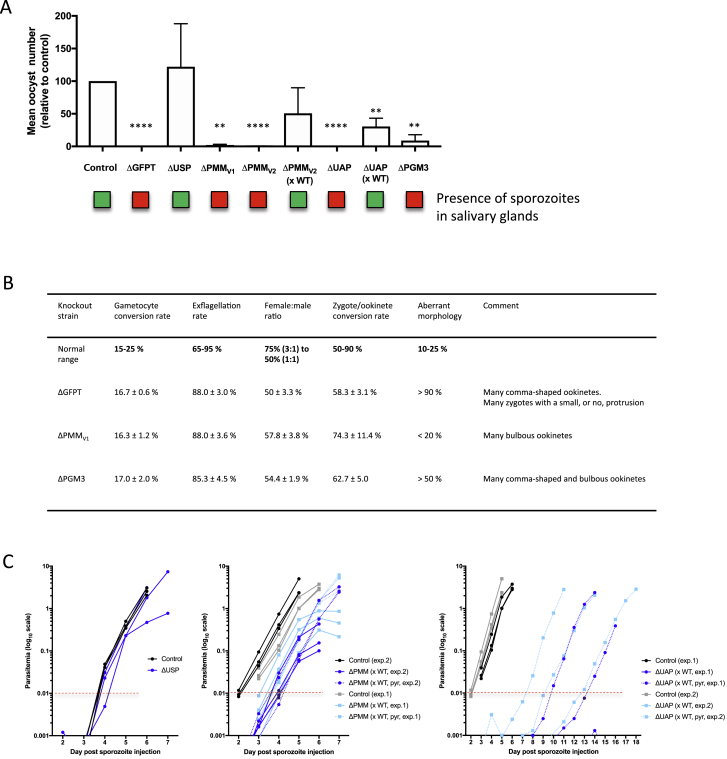
Phenotypic Analysis of Gene Knockout Mutant Parasite Lines Associated with the Amino Sugar Biosynthesis Pathway, Related to Figure 7 (A) Graph showing oocyst numbers (relative to control; set to 100%) at day 7 post infection for ΔUSP, ΔPMMv1, ΔPMMv2, ΔPMMv2 (xWT), ΔUAP, ΔUAP (xWT) and ΔPGM3 parasites. Error bars indicate standard deviation. The results were statistically evaluated by a one-way analysis of variance (ANOVA) test with Dunnet’s multiple comparisons (^∗∗^p < 0.01, ^∗∗∗^p < 0.001, ^∗∗∗∗^p < 0.0001). Boxes shown below the graph indicate the presence (green) or absence (red) of sporozoites from the salivary glands for each knockout strain between days 18 and 21 post-infection. (B) Table displaying sexual and mosquito stage phenotypic data for ΔGFPT, ΔPMMv1 and ΔPGM3 parasites; gametocyte conversion rate, exflagellation rate, female: male ratio, zygote to ookinete conversion rate and percentage of ookinetes showing abnormal morphology. Data is shown in relation to normal ranges for such phenotypic assessments. (C) Graphs to show parasitemia for mice injected with ΔUSP parasites and ΔPMMv2 (xWT) and ΔUAP (xWT) parasites (based on FACS). Data from all mice (control and KOs) are shown for two independent experiments and lines are drawn for the relative light unit level or parasitemia considered as being the point at which a mouse has become positive.

We examined the specific role of FAE at the SG-B2 transition using single knockout mutants (Figures S3 and 6B; Table S6). ΔELO-A parasites were characterized by normal mosquito development (Figure S5B), but liver stages showed a drastically reduced size at 48 hpi *in vitro* and failed to mature to the point of host cell detachment (Figures 6C and 6D). *In vivo*, only two of the six mice injected with 5,000 ΔELO-A sporozoites became infected, and those that did had a pre-patent period extended by 7 days (Figures 6E and S5E). While these data demonstrate the importance of FAE for liver-stage maturation, other FAE genes have essential roles during mosquito-stage development. DEH of *P. berghei* is important for mosquito-stage development (Guttery et al., 2014), and two ΔKCR mutants generated here (ΔKCR_V1_, ΔKCR_V2_) also showed a complete growth arrest in the mosquito. Ookinetes and oocysts were produced in normal numbers (Figures S5B and S5D), but oocysts gradually disappeared over the course of development and failed to give rise to sporozoites (Figures S5B and S5C). We successfully crossed ΔKCR_V2_ with wild-type parasites to study ΔKCR_V2_ sporozoites emerging from a heterozygous oocyst (Figure S5B) and found that ΔKCR_V2_ produced significantly smaller liver stages at 48 hpi *in vitro* (Figure 6C), which failed to fully mature (Figure 6D). *In vivo*, only 4 of 9 mice injected with ΔKCR_V2_ sporozoites became infected, with a delay in pre-patency of 6.7 days (Figures 6E and S5E), confirming the strong drop for KCR in the screen.

PhenoMapping with the iPbe model suggests that FAE genes become essential when long-chain fatty acids (such as [9Z]-octadecenoic acid) and unsaturated fatty acids (such as linoleate) cannot be scavenged at a sufficient rate. Long-chain fatty acids have diverse roles in protein trafficking, synthesis of cellular structures, and signaling. Some of these functions are also likely important in blood stages, which we hypothesize obtain sufficient long-chain fatty acids from the host red blood cell or serum (Mi-Ichi et al., 2006).

The ER also accommodates an acyl-CoA desaturase (SCD; Gratraud et al., 2009) to generate unsaturated fatty acids and a pathway to incorporate them into glycerophospholipids (Figure 6A; Table S4) (Ramakrishnan et al., 2013). SCD consumes ferrocytochrome b5, which needs to be recycled. The iPbe model includes a putative cytochrome-b5 oxidoreductase (CBR) as candidate for this function (Figure 6A). CBR is >400-fold depleted at the SG-B2 transition, and an individual ΔCBR mutant is characterized by smaller liver stages at 48 hpi *in vitro* (Figure 6C), a 50% reduction in detached cell formation (Figure 6D), and a delay in pre-patent period by 2.7 days after sporozoite injection (Figure 6E), corresponding to a liver-stage load that is reduced by more than 90%. iPbe-liver suggests that the functions of SCD and CBR in the ER are coupled, and hence SCD, which was not covered in the screen, might also be essential in the liver stages of *Plasmodium* infection. In other eukaryotic cells, SCD is part of a multiprotein complex, along with cytochrome b5 and CBR (Mi-Ichi et al., 2006), a feature that we hypothesize extends to *P. berghei*. Taken together, our screen, iPbe-liver, and the analyses of ΔELO-A, ΔKCR, and ΔCBR mutants show for the first time the strong dependence of the *Plasmodium* liver-stage parasite on FAE.

### Amino Sugar Biosynthesis Is Important for Both Mosquito- and Liver-Stage Development

**Figure 7:**
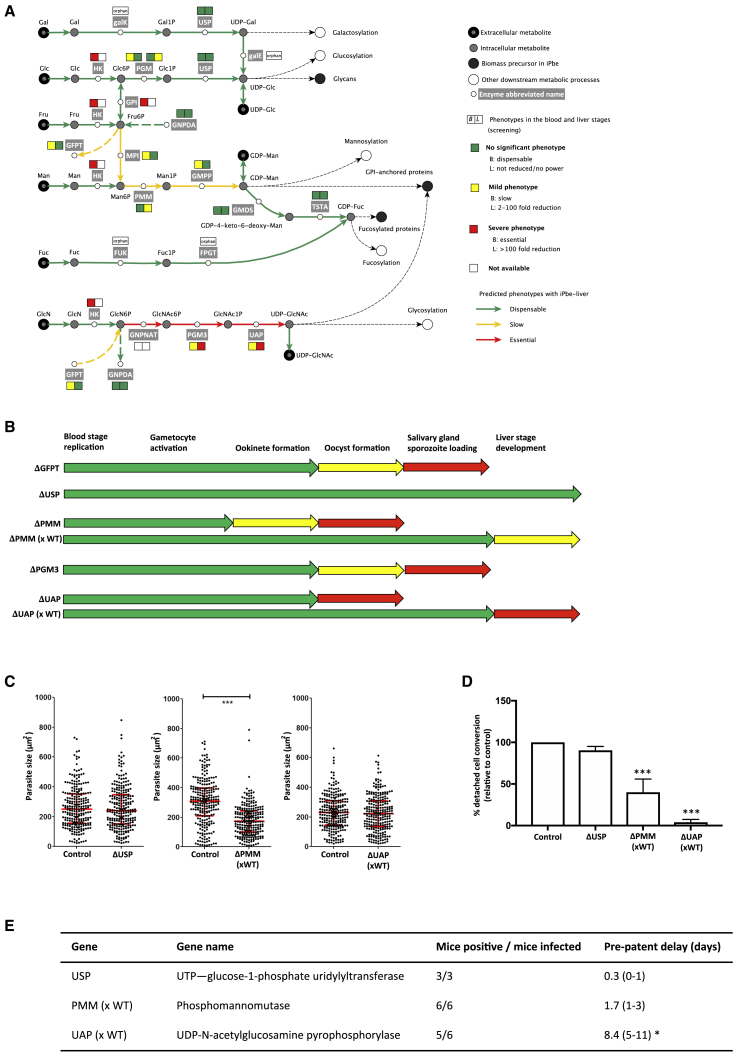
Mutations in Amino Sugar Metabolism Disrupt Liver-Stage Development (A) Activation of sugars in the *Plasmodium* cytosol based on iPbe. See Table S4 for gene IDs, enzyme functions, and reactions. Gal, D-galactose; Gal1P, D-galactose 1-phosphate; UDP-Gal, UDP-d-galactose; Glc, D-glucose; Glc6P, D-glucose 6-phosphate; Glc1P, D-glucose 1-phosphate; UPD-Glc, UDP-glucose; Fru, D-fructose; Fru6P, D-fructose 6-phosphate; Man, D-mannose; Man6P, D-mannose 6-phosphate; Man1P, D-mannose 1-phosphate; GDP-Man, GDP-mannose; GDP-4-keto-6-deoxy-Man, GDP-4-dehydro-6-deoxy-d-mannose; GDP-Fuc, GDP-L-fucose; Fuc, 6-deoxy-L-galactose/fucose; Fuc1P, L-fucose 1-phosphate; GlcN, D-glucosamine; GlcN6P, D-glucosamine 6-phosphate; GlcNAc6P, N-acetyl-d-glucosamine 6-phosphate; GlcNAc1P, N-acetyl-alpha-d-glucosamine 1-phosphate; UDP-GlcNAc, UDP-N-acetyl-d-glucosamine. (B) Schematic representation of developmental phenotypes of single KOs and mutants from ΔPMM × WT and ΔUAP × WT genetic crosses. Green, phenotype not significantly different from WT. Yellow, significantly reduced. Red, developmental block, except for liver stage, where red indicates phenotype significantly different from WT (>2-day delay in pre-patent period). (C) Size of 250 cultured EEFs 48 hpi; median and interquartile ranges in red. ^∗∗∗^p < 0.001; ^∗^p < 0.05 by Kruskal-Wallis test. (D) Relative maturation of EEFs measured as conversion of infected host cells to detached cells at 48 hpi. Error bars show standard deviations from 8 biological replicates. The results were statistically evaluated by a one-way analysis of variance (ANOVA) test with Dunnet’s multiple comparisons (^∗∗∗^p ≤ 0.001). (E) Overall transmission success given as the number of mice that became blood stage positive after injection of 5,000 sporozoites and the mean delay (range) in pre-patency compared to mice infected with WT. ^∗^, gene KO parasites with a significantly “slow” blood stage phenotype (Bushell et al., 2017). See Figure S6C for plots showing the course of blood stage infections after sporozoite injection. Supplemental Information

*Plasmodium* parasites use a canonical metabolic pathway to activate sugars such as glucose, mannose, galactose, fructose, fucose, and glucosamine (Figure 7A; Table S4), and these nucleotide sugars serve to produce glycoconjugates like glycosylphosphatidylinositol (GPI)-ancho red proteins, which are the primary form of cell-surface proteins in protozoa. In pathogens like *Trypanosoma brucei* and *Plasmodium* spp., GPI-anchored proteins play a role in invasion (Sanders et al., 2006), signaling (Gazzinelli et al., 2014), and endocytosis (Overath and Engstler, 2004). While the biosynthesis of nucleotide sugars in *P. berghei* blood stages was shown to be dispensable, the parasite critically relies on the production of GPI anchors (Bushell et al., 2017). We hence wondered what could be an alternative source of nucleotide sugars to the *Plasmodium* parasites.

**Figure S7:**
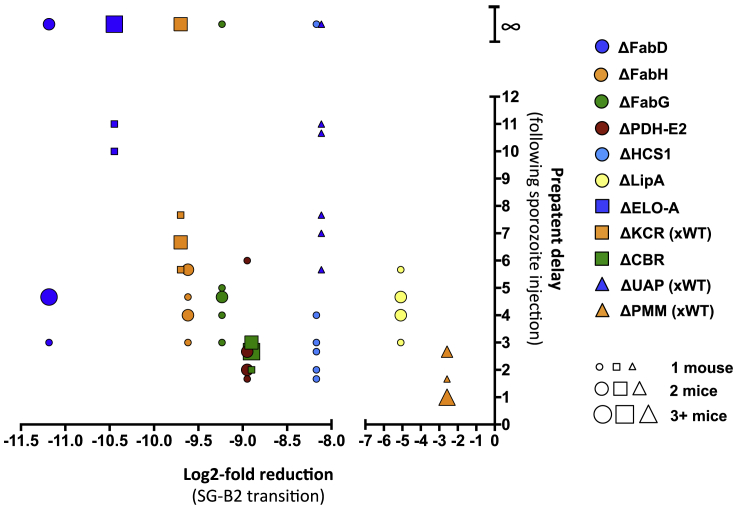
A Graph Showing Correlation between the SG-B2 Transition Phenotype Seen in the M-L Screen and In Vivo Data for Single Gene Knockout Parasite Lines, Related to Figures 5, 6, and 7 The prepatent delay in the appearance of blood stage infection following injection of sporozoites is shown for each mouse injected with single gene knockout parasite lines ΔFabD, ΔFabH, ΔFabG, ΔPDH-E2, ΔHCS1, ΔLipA, ΔELO-A, ΔKCR (xWT), ΔCBR, ΔUAP (xWT) and ΔPMM (xWT) in relation to the log2-fold reductions at the SG-B2 transition in the M-L screen. The sizes of data point indicate the number of mice (total of 6) that show each pre-patent period. The ∞ symbol indicates mice that never became positive after sporozoite injection.

The screen revealed strong and surprising SG-B2 phenotypes within the network of pathways that lead to activation of sugars (Figure 7A; Table S5). Genes encoding enzymes involved in the biosynthesis of UDP-N-acetyl glucosamine, PGM3, and UAP showed log2-fold reductions of −7.8 and −8.1, respectively. To investigate the role of enzymes involved in the biosynthesis of UDP-N-acetyl glucosamine, we generated the single knockout mutants ΔPGM3 and ΔUAP (Figures S3 and 7B; Table S6). Both mutants had a strong mosquito-stage phenotype, with ΔUAP parasites failing to produce oocysts (Figure S6A) and ΔPGM3 parasites producing a relatively high percentage of ookinetes with an aberrant morphology (Figure S6B) and reduced number of oocysts compared to wild type parasites; no sporozoites were observed (Figures 7B and S6A). ΔUAP parasites were crossed with WT parasites during blood-stage development, and using these sporozoites, a strong liver-stage phenotype was observed both *in vitro* and *in vivo*. Although the size of liver stages *in vitro* at 48 h was similar to that of wild-type parasites, only very few detached cells were formed (Figures 7C and 7D), and *in vivo* the pre-patent period was prolonged by 6 days (Figures 7E and S6C). These observations confirmed the phenotype seen for UAP in the screen, and PhenoMapping of iPbe revealed the biosynthesis of N-acetyl-glucosamine to be of key importance in liver-stage parasite development.

Other genes involved in sugar activation showed only mild or no SG-B2 screen phenotypes; USP and GFPT showed no significance at the SG-B2 transition, and PMM showed only a mild SG-B2 phenotype (log2FC = −2.6). We produced single knockout mutants for each of these genes (Figures S3 and 7B; Table S6). ΔUSP parasites, lacking the USP enzyme involved in production of UDP-Glc, showed no phenotype at any life-cycle stage, including the liver stage (Figures 7B–7E, S6A, and S6C), confirming the lack of phenotype seen in the screen. Two ΔPMM mutants (ΔPMM_V1_ and ΔPMM_V2_), lacking the PPM enzyme involved in the production of GDP-mannose, showed strongly reduced oocyst numbers, despite normal levels of ookinete formation, and these parasites failed to produce salivary gland sporozoites (Figures S6A and S6B). When crossed with wild-type parasites, ΔPMM_V2_ sporozoites showed only a slight delay in pre-patent period when injected into mice (Figures 7E and S6C) and showed only a mild defect during *in vitro* liver-stage development (Figure 7D), in agreement with the mild liver-stage phenotype observed in the screen and also with the lack of a phenotype for the gene encoding GMPP, which catalyzes a different step in the same pathway toward the production of GDP-mannose. The activation of glucosamine and fructose is linked through glutamine—fructose-6-phosphate aminotransferase (GFPT). ΔGFPT parasites showed a strong phenotype in the formation of ookinetes (Figure S6B), with a large percentage of ookinetes showing an aberrant morphology. ΔGFPT parasites failed to produce oocysts (Figure S6A), preventing downstream liver-stage analysis.

PhenoMapping with iPbe liver and blood predicts that the parasite can take up nucleotide sugars from the host cells (both the erythrocytes and hepatocytes), and a difference between the uptake rate or availability of the different nucleotide sugars from the host would explain the differences observed between blood- and liver-stage phenotypes (Table S3). This analysis indicates that UDP-N-acetyl-D-glucosamine is the most limiting nucleotide sugar for liver-stage development, while GDP-mannose and UDP-glucose are accessible in higher amounts from the host cell.

All genes that had been shown to have a more than 100-fold reduction in abundance in the screen showed a clear delay in pre-patent period when sporozoites of single knockout mutants were injected into mice (Figures S7, 5E, 6E, and 7E). Taken together, the liver-stage phenotypes of single gene knockout mutants matched well the phenotypes seen for the SG-B transition of the M-L screen (Figure S7).

## Discussion

To fill the critical knowledge gap of *Plasmodium* mosquito- and liver-stage metabolic pathways, we performed the first systems-level analysis of *Plasmodium* pre-erythrocytic physiology by combining large-scale genetic screening with metabolic modeling. Barseq sequencing enabled us to analyze the phenotypes of >1,300 gene knockout parasites at three developmental transitions, namely (1) between blood stages and mosquitoes, (2) between the mosquito midgut and the salivary glands, and (3) as the parasites transitioned from the mosquito salivary gland to establish and undergo development within the liver. Although in the polyploid stages cross-fertilization leading to heterozygosity imposes limits on the power of pooled screens, we find that barcoded knockout alleles can be effectively transmitted through population bottlenecks to the haploid sporozoite stage in the salivary gland, allowing mutants to be screened for the first time at a significant scale for gene functions during subsequent development.

Our ability to predict the complement of liver-stage essential genes from the screen is limited by three factors: (1) the current genetic system for *P. berghei*, which does not allow blood-stage essential genes to be screened at other life cycle stages, (2) the coverage of the *Plasmo*GEM resource, which extends to only around 60% of the core genome, and (3) the inherent error rate of any genetic screen. To compensate for these challenges, we developed an iPbe-liver metabolic model that integrates data from the current screen with available genomic, transcriptomic, and metabolomic data to predict with good accuracy the phenotypes associated with a majority of the metabolic subsystems, such as fatty acid and amino sugar biosynthesis. The overall Matthew correlation coefficient of our blood- and liver-stage models (0.51 and 0.70, respectively) is similar to MCC scores of 0.6 achieved for model organisms *E. coli* (Monk et al., 2017) and *S. cerevisiae* (Heavner and Price, 2015). These considerations, together with our success in validating all attempted phenotypes with cloned mutants, demonstrate that iPbe-liver and iPbe-blood are valid platforms for the study of liver- and blood-stage metabolic functions and therefore drug target prioritization.

Our discoveries that amino sugar metabolism and FAE are important for the *Plasmodium* liver stage illustrate how the combined use of metabolic modeling and high-throughput gene knockout screening can generate deep new insights into a poorly tractable organism. iPbe-liver will guide the design of future gene knockout experiments and analysis of metabolic capabilities in the liver stages for the 141 genes predicted to be essential at this stage but for which single knockout parasites have not been generated or phenotyped. Moreover, iPbe-liver predicts liver-stage-specific essentiality for 27 genes that are fully dispensable in iPbe-blood (Table S3). These genes belong to seven metabolic subsystems related to fatty acid, TCA, amino acid, heme, lipoate, and shikimate metabolism, and their metabolic function might be associated with the antimalarial activity of compounds acting in the pre-erythrocytic stages (Antonova-Koch et al., 2018). Further validation and curation of the iPbe-liver model will lead to an even deeper understanding of liver-stage-specific *Plasmodium* metabolism. Possible avenues include (1) further screens as the *Plasmo*GEM resource grows to cover more of the remaining 32% of 428 genes in the model that currently lack vectors, (2) screens for synthetic lethal interactions predicted by the model, which are now possible in *P. berghei* (Fang et al., 2018), or (3) scaling up conditional knockout approaches to study at the liver stage the large number of blood-stage-essential genes.

Validating the screen and model with individual mutants confirmed the crucial role of the FASII pathway for liver stages and further revealed the essentiality of the FAE pathway for liver-stage parasites and its coupled enzymes, e.g., CBR in the ER, demonstrating a clear dependence of the liver stage on its own fatty acid metabolism. Through modeling genomic data, we show here for the first time that not only short-chain fatty acids but also long-chain and unsaturated fatty acids are insufficient to support the rapid *Plasmodium* growth in the liver stages.

We have also demonstrated for the first time the critical role of amino sugar biosynthesis in the mosquito and liver stages of *P. berghei.* We hypothesize that the parasite has a differential ability to take up from the host sufficient quantities of specific amino sugars, with the uptake of N-acetyl-glucosamine appearing to be significantly limited in the liver stage. This would to our knowledge be the first indication of an intracellular pathogen taking up amino sugars from its host cell. Despite the biosynthesis pathway of amino sugars being identical in *P. falciparum* and *P. berghei*, the amino sugar metabolism of blood stages appears to differ between these malaria parasites. Data from phenotyping of blood stages of knockout mutants (Bushell et al., 2017) for genes encoding UAP or PGM3 show that the synthesis of UDP-N-acetyl-glucosamine is not essential for blood stage fitness of *P. berghei*. However, in *P. falciparum*, unsuccessful attempts to knock out GNPNAT (Cova et al., 2018) and the underrepresentation of transposon insertions into GNPNAT, PGM3, and UAP genes (Zhang et al., 2018) indicate that the biosynthesis of UDP-N-acetyl-glucosamine is essential for *P. falciparum* blood-stage growth. We hypothesize that the difference in blood-stage essentiality of amino sugar metabolism between *P. falciparum and P. berghei* is related to the nutrient availability from the host cells in which these parasites replicate during the erythrocytic stage. *P. berghei* parasites preferentially replicate in reticulocytes, which show a considerably higher level of UDP-N-acetyl-glucosamine than normocytes, the host cell of cultured blood-stage *P. falciparum* (Srivastava et al., 2016). We suggest that the *P. berghei* blood-stage parasite does not rely entirely on its own synthesis of UDP-N-acetyl-glucosamine, as it can acquire sufficient quantities from the reticulocyte. However, it seems that acquisition from the host environment is insufficient in the mosquito and liver stages, rendering this pathway essential at these stages. To date, USP is the only known *Plasmodium* enzyme able to produce UDP-glucose and UDP-galactose, and it is non-essential in both *P. berghei* and *P. falciparum* at all stages investigated (Bushell et al., 2017, Zhang et al., 2018). However, UDP-glucose is required to produce glycans, suggesting uptake of extracellular UDP-glucose. Alternative explanations for the dispensability of USP in all life-cycle stages would be the presence of an as yet uncharacterized isoenzyme of USP or promiscuity of UAP to catalyze the USP-associated reactions.

Targeting *Plasmodium* liver-stage parasites selectively is emerging as an attractive alternative strategy in the face of emerging resistance to the latest frontline combination therapies against blood stages of the parasite. Potent starting points for developing a liver-stage-specific compound have recently been discovered in screens using *P. berghei* (Antonova-Koch et al., 2018). Identifying their targets is now an important next step to enable target-led chemical optimization of candidate compounds toward new prophylactic drugs. The combined experimental and computational analysis of pre-erythrocytic development and liver-stage metabolism has led us to identify seven additional metabolic subsystems that become essential compared with the blood stage, which provides a rational basis for the future design of antimalarial therapies targeting metabolic proteins.

## STAR★Methods

### Key Resources Table

**Table:** 

REAGENT or RESOURCE	SOURCE	IDENTIFIER
**Critical Commercial Assays**		

Amaxa P3 Primary Cell 4D-Nucleofector X Kit S	Lonza	V4XP-3032
4D-Nucleofector Core Unit	Lonza	AAF-1002B
4D-Nucleofector X Unit	Lonza	AAF-1002X
MiSeq Reagent Kit v2 (300-cycles)	Illumina	MS-102-2002
MiSeq Sequencing System	Illumina	N/A
QuickExtract DNA Extraction Solution	Lucigen	QE09050
Cell Culture Lysis Reagent	Promega	E1531
Luciferase Assay Reagent	Promega	E1500

**Deposited Data**		

*P. berghei* relative growth rate phenotypes in blood stages	Bushell et al., 2017	https://doi.org/10.1016/j.cell.2017.06.030
*P. berghei RNA-seq data in blood stages*	Otto et al., 2014	https://doi.org/10.1186/s12915-014-0086-0
*P. berghei RNA-seq data in liver stages*	Caldelari et al., 2019	https://doi.org/10.1186/s12936-019-2968-7
*Compiled metabolomics dataset from P. falciparum*	Chiappino-Pepe et al., 2017a	https://doi.org/10.1371/journal.pcbi.1005397
*P. berghei ANKA protein sequences (fasta file)*	PlasmoDB	Release 26
*K-orthology groups sequences for enzymatic functional annotation*	KEGG database	2015
*Genome-scale metabolic model of P. falciparum iPfa*	Chiappino-Pepe et al., 2017a	https://doi.org/10.1371/journal.pcbi.1005397
*P. falciparum 3D7 protein sequences (fasta file)*	PlasmoDB	Release 11.1
*The ATLAS of Biochemistry*	https://lcsb-databases.epfl.ch/atlas/	2018
*Metabolic reactions in the malaria parasites*	MPMP database	2016-2018
*Reactions producing iron sulfur clusters*	iJO1366	N/A

**Experimental Models: Cell Lines**		

Arrayed library of *E. coli* TSA cells harboring linear plasmids containing *P. berghei* gene targeting vectors.	*Plasmo*GEM resource	https://plasmogem.sanger.ac.uk/search
HeLa cells	ECACC	93021013

**Experimental Models: Organisms/Strains**		

Rat: RCC Han Wistar outbred (female)	Envigo+++	RccHan:WIST
Mouse: BALB/c inbred (female)	WTSI & Envigo & Janvier	BALB/cOlaHsd
Mouse: C57BL/6JRj	Janvier	C57BL/6JRj
Mouse: OF1 mice	Charles River	OF1, 612
*P. berghei:* ANKA cl15cy1 wild-type parasites	N/A	cl15cy1
*PbANKA-mCherry*_*hsp70*_*+Luc*_*eef1α*_*(1868cl1)*	Prado et al., 2015	www.pberghei.eu (RMgm-1320)
*PbmCherry*	Burda et al., 2015	www.pberghei.eu (RMgm-928)
*E. coli:* BigEasy-TSA	Lucigen	60224
*Anopheles stephensi* mosquitoes, STE2	MR4, BEI resources	MRA-128

**Software and Algorithms**		

TEX-FBA	www.github.com/EPFL-LCSB/texfba	1.0
matTFA	www.github.com/EPFL-LCSB/matTFA	1.0
MATLAB	Mathworks	R2016a and R2017a
CPLEX	IBM	12.71
COBRA Toolbox (used updated version within matTFA)	https://opencobra.github.io/cobratoolbox/stable/	2.0
RAVEN Toolbox	https://github.com/SysBioChalmers/RAVEN	1.08
The R Project for Statistical Computing	https://www.r-project.org/	3.5.0
Tidyverse	https://www.tidyverse.org/	1.2.1
TargetP	http://www.cbs.dtu.dk/services/TargetP/	1.1
MitoProtII	https://ihg.gsf.de/ihg/mitoprot.html	1.101
ApicoAP	https://bitbucket.org/wsu_bcb/apicoap/src/master/	2.0

### Lead Contact and Materials Availability

Further information and requests for resources and reagents should be directed to and will be fulfilled by the Lead Contact, Volker Heussler (volker.heussler@izb.unibe.ch).

*Plasmo*GEM vectors are available from https://plasmogem.sanger.ac.uk. *Plasmodium berghei* parasite lines generated in this study will be available from the Leiden Malaria Research Group at the LUMC; https://www.lumc.nl/org/parasitologie/research/malaria/transgenics-mutants-berghei/.

### Experimental Models and Subject Details

#### Use of rodents at Wellcome Sanger Institute

All animal research was conducted under licenses from the UK Home Office, and protocols were approved by the Animal Welfare and Ethical Review Body of the Wellcome Sanger Institute. Rodents were kept in specific-pathogen-free conditions and subjected to regular pathogen monitoring by sentinel screening. They were housed in individually ventilated cages furnished with autoclaved aspen woodchip, fun tunnel and Nestlets at 21 ± 2°C under a 12:12 h light-dark cycle at a relative humidity of 55 ± 10%. They were fed a commercially prepared autoclaved dry rodent diet and water, both available *ad libitum*. The health of animals was monitored by routine daily visual health checks. The parasitemia of infected animals was determined by methanol fixed and Giemsa-stained thin blood smears.

Female RCC Han Wistar outbred rats (Envigo, UK) aged eight to fourteen weeks were infected with *P. berghei* parasites by intraperitoneal injection. Infected rats served as donors for *ex vivo* schizont cultures typically on day four to five of infection, at a parasitemia of 1%–5%. Rats were housed as two cage companions. Rats were terminally anaesthetized by vaporized isoflurane administered by inhalation prior to terminal bleed. Rats were used because they give rise to more schizonts with higher transfection efficiency compared to mice. Transfection efficiency is critical when screening pools of vectors.

Mice were bred at the Wellcome Sanger Institute or purchased from Envigo. Transfected parasites were injected intravenously into the tail of female adult BALB/c inbred mice aged 8-22 weeks (median age 10 weeks). This animal model was chosen to minimize host genetic variability and to obtain robust infections with a low incidence of cerebral pathology. Experimental groups consisted of three mice housed together. Three internally controlled biological replicates per parasite pool proved adequate to identify phenotypes with confidence.

#### Use of rodents at University of Bern

The work in Bern (Switzerland) was performed experiments in accordance with the guidelines of the Swiss Tierschutzgesetz (TSchG; Animal Rights Laws) and approved by the ethical committee of the University of Bern (Permit Number: BE132/16). Mice were kept in specific-pathogen-free conditions and subjected to regular pathogen monitoring by sentinel screening. They were housed in individually ventilated cages furnished with autoclaved aspen woodchip, a mouse house and paper tissue at 21 ± 2°C under a 12:12 h light-dark cycle at a relative humidity of 55 ± 10%. They were fed a commercially prepared autoclaved dry rodent diet and water, both available *ad libitum*. The health of animals was monitored by routine daily visual health checks. The parasitemia of infected animals was determined by FACS analysis.

Female BALB/c mice (6-8 weeks; Janvier laboratories, France) were used to maintain transfected parasites and for feeding of mosquitoes with parasites. Mice were injected via an intraperitoneal or intravenous route. When parasitemia reached 2%–5%, mice were euthanized in a CO_2_ chamber and parasites isolated following exsanguination. For feeding of mosquitoes, upon reaching a parasitemia of 7%–15%, mice were anaesthetized with a terminal dose of ketamine:xylazine and when no longer reacting to touch stimulus were placed on a cage of approximately 150 mosquitoes. For *in vivo* liver experiments, female C57BL/6JRj mice (6-8 weeks; Janvier laboratories, France) were injected with sporozoites and were euthanized when parasitemia reached 2% to prevent any incidence of cerebal malaria.

#### Use of rodents at Leiden University Medical Centre

All experiments were approved by the Animal Experiments Committee of the Leiden University Medical Center (DEC 12042 and 14207). The Dutch Experiments on Animal Act is established under European guidelines (EU directive no. 86/609/EEC regarding the Protection of Animals used for Experimental and Other Scientific Purposes). Mice were kept in specific-pathogen-free conditions and subjected to regular pathogen monitoring by sentinel screening. Mice were housed in individually ventilated cages furnished with autoclaved aspen woodchip, fun tunnel, a wood chew block and Nestlets at 21 ± 2°C under a 12:12 h light-dark cycle at a relative humidity of 55 ± 10%. They were fed a commercially prepared autoclaved dry rodent diet and water, both available *ad libitum*. The health of animals was monitored by routine daily visual health checks. The parasitemia of infected animals was determined by methanol fixed and Giemsa-stained thin blood smears. Transfection and phenotypic analysis were performed with female OF1 mice (6-7 weeks; Charles River Laboratories, France).

#### Use of mosquitoes at the University of Bern

Mosquitoes used at the University of Bern were of the strain *Anopheles stephensi*. They were bred in-house in conditions of 27°C, 80% humidity. Following infection with *P. berghei* parasites, mosquitoes were maintained at 20.5°C at 80% humidity. All mosquitoes were supplied with 8% fructose solution (filter sterilized, supplemented with PABA) and for dissection were anaesthetized in chloroform vapor before submersion in 70% alcohol.

### Method Details

#### M-L screening using *Plasmo*GEM vectors

##### Generation of transgenic parasite pools

*P. berghei* (*Pb*mCherry, Burda et al., 2015) schizonts for transfection of vector pools were produced in female Wistar Han rats (150-200 g, Envigo, UK) to achieve maximal transfection efficiency. To generate pools of mutants for phenotyping by barcode counting, equal amounts of 50-60 *Plasmo*GEM vectors were combined and the mixture digested with *Not*I to release the targeting vectors from the bacterial vector backbone and purified by standard ethanol precipitation before resuspension in dH_2_0. A total of 5-6 μg of the digested vector mix in a volume of 10 μL dH_2_0, typically containing 100 ng of DNA for each vector, was used per transfection. For each vector pool, DNA was prepared in triplicate in a single digest, typically 15-18 μg DNA in 30 μL dH_2_0. Experiments with single vectors used 2 μg of *Not*I-restricted DNA per transfection. *Plasmo*GEM identification numbers for vectors used in this study are listed in Table S7. Transfections, at Wellcome Sanger Institute, were done by electroporation of purified rat schizonts as described (Janse et al., 2006), with modifications for pooled transfection methodology (Bushell et al., 2017). Briefly, parasites for schizont culture were obtained from female Wistar Han rats to achieve maximal transfection efficiency and were cultured for 22-24 h before schizonts were isolated on a 55% Nycodenz/PBS cushion. Isolated schizonts were washed in complete media and electroporated using the 4D Nucleofector System (Lonza) in 16-well strips according to the pulse program FI-115. Transfected schizonts were injected intravenously into three separate BALB/c mice. Resistant parasites were selected by pyrimethamine (70 μg/L in the drinking water). Infections were monitored daily using Giemsa-stained thin blood films.

##### Transmission of transgenic parasite pools

When parasitemia reached ∼1.0%–10%, typically day 6 post-transfection, parasites were harvested by heart puncture. Infected blood was immediately shipped at 4°C to the University of Bern in complete schizont medium (Janse et al., 2006), where it was transferred to two phenylhydrazine-treated BALB/c mice by intravenous injection upon arrival. When the parasitemia of infected mice reached at least 7%, a blood sample was taken and mice were anaesthetized and used to feed female *A. stephensi* mosquitoes. At day 15 and day 22 post-infection, midguts and salivary glands, respectively, were removed from more than 30 mosquitoes. At day 22 post-infection, sporozoites from 30-40 infected mosquitoes were intravenously injected into two mice and blood was a blood sample was taken 5 days post-injection.

#### gDNA sampling and Illumina sequencing

The parasite genomic DNA (gDNA) isolation from blood was performed using phenol-chloroform extraction as described in (Gomes et al., 2015). A total of 30 μL of infected blood was collected from the tail on days 7 post-transfection and diluted in 200 μL of phosphate-buffered saline. Total DNA was extracted from each sample and resuspended in 50 μL water. gDNA isolation from oocyst and salivary gland sporozoites was performed by using the QuickExtract DNA Extraction Solution (QE) from Epicenter. Briefly, thirty mosquito midguts or homogenized salivary glands, in PBS or MEM respectively, were centrifuged at 500 g for 3 min and resuspended in 100 μL (midguts) or 50 μL (salivary glands) QE buffer. Tubes were transferred to 65°C (slow shaking) and incubated for 6 min and transferred to 98°C for 2 min. The gDNA was then stored at 4°C for further analysis. For sampling from blood of sporozoite-injected mice, 300 μL blood was taken five days post-sporozoite injection and processed as above.

To sequence the vector-specific barcodes, 0.1-10 μL of each DNA sample served as a template for a PCR reaction using Advantage 2 Taq polymerase (Clontech) with primers arg444 and arg445 (1 × 95°C/5 min denaturation, 35 × 95°C/30 s, 55°C/20 s, 68°C/8 s, 1 × 10 min at 68°C), which bind to constant annealing sites flanking each barcode. For sample-specific indexing, 5 μL of the first amplicon served as template for a further ten amplification cycles (1 × 95°C/2 min, 10 × 95°C/30 s, 68°C/15 s, 1 × 5 min, 68°C) using one generic oligonucleotide (PE1.0) and one of a set of 64 sample-specific indexing oligonucleotides (Table S7). A total of 100 ng of each sequencing library was pooled and quality controlled by quantitative PCR for the presence of sequencing adaptors. Libraries were sequenced using MiSeq Reagent Kit v2 (300 cycles) from Illumina (MS-102-2002). Due to their low complexity, PCR amplicon libraries had to be diluted to 4 nM before loading the flow cell of a MiSeq instrument (Illumina) at low cluster density (4 × 105 clusters/mm2) with 30%–50% of PhiX spike-in. Sequencing of 150 bp paired-ends yielded 1.0–1.5 × 105 reads on average for each of the 32 samples. Using a Perl script, barcode sequences were extracted from sequencer output, counted, and the relative abundance of each barcode within the pool determined. The quantitation was considered reliable for barcodes accounting for at least 0.1% of all counts.

#### Generation of single KO parasite lines

##### Transfection to generate gene-deletion mutants

Single gene knockout *P. berghei* parasites were generated for a total of 20 genes using deletion plasmids obtained from *Plasmo*GEM (Wellcome Sanger Institute, UK, http://plasmogem.sanger.ac.uk/ (Gomes et al., 2015, Schwach et al., 2015)). The parental *P. berghei* ANKA parasite line to transfect 3xHA-hdhfr-yFCU vector was used: 1868cl1 (*Pb*ANKA-mCherry_hsp70_+Luc_eef1α_; line RMgm-1320; www.pberghei.eu; (Prado et al., 2015)) which contains the *mcherry* gene under control of the strong *hsp70* promoter and *luciferase* gene under control of the constitutive *eef1α* promoter integrated into the neutral *230p* gene locus (PBANKA_0306000). The parental *P. berghei* ANKA line used for transfection of GOMO-GFP-Cherry-FACS vectors was cl15cy1 (Janse et al., 1989). Parasites of these lines do not contain a drug-selectable marker in their genome.

For genes PDH-E2 (PBANKA_0505000), HCS1 (PBANKA_0511000), FabD (PBANKA_1410500), FabG (PBANKA_0823800), FabH (PBANKA_0308200), LipA (PBANKA_1357500), ELO-A (PBANKA_0820900), KCR (PBANKA_0522400), CBR (PBANKA_1143400), USP (PBANKA_1232300), PMM (PBANKA_0501700), PGM3 (PBANKA_0918200), GFPT (PBANKA_0509300), LPD1 (PBANKA_1446900), IS-SDHA (PBANKA_1428800) and GCH1 (PBANKA_1438900), single knockout parasites were generated using a vector with a “3xHA-hdhfr-yFCU” selection cassette, which contains a *hdhfr::yfcu* selectable marker (SM), under the control of the *P. berghei eef1α* promoter region and 3′ terminal sequence of *pbdhfr*. In the cases of KCR and PMM, these sKO lines are sometimes referred to as version 1 (ΔKCR_v1_, ΔPMM_v1_).

For genes KCR (PBANKA_0522400), PMM (PBANKA_0501700) and UAP (PBANKA_1356600), single knockout parasite lines were generated using a vector with a “GOMO-GFP-Cherry-FACS” selection cassette, which contains not only the *hdhfr::yfcu* selectable marker (SM), under the control of the *P. berghei eef1α* promoter region and 3′ terminal sequence of *pbdhfr* but additionally cassettes for GFP and mCherry expression, controlled by the *hsp70* and *eef1α* promoters respectively and terminated by the 3′ terminal sequence of *pbdhfr*. In the cases of KCR and PMM, these sKO lines are sometimes referred to as version 2 (ΔKCR_v2_, ΔPMM_v2_). The GOMO cassette was adapted for use with *Plasmo*GEM vectors in collaboration with Olivier Silvie and adapted from (Manzoni et al., 2014).

Before transfection, constructs were linearized by digesting with *Not*I. Parasites of line 1868cl1 or cl15cy1 were transfected with the 3xHA-hdhdfr-yFCU or GOMO-GFP-Cherry-FACS constructs respectively using standard transfection technologies and transformed parasites selected by positive selection with pyrimethamine (Janse et al., 2006).

Parasites harboring a 3xHA-hdhfr-yFCU cassette were cloned by limiting dilution. Parasites harboring a GOMO-GFP-Cherry-FACS cassette were enriched by FACS sorting for red/green parasites, using a procedure based on that of (Manzoni et al., 2014); once the parasitemia reached 0.1%–1%, a drop of blood was taken from the mouse tail and diluted with 500 μL of Serum-free/Phenol-red free William’s E-Medium (WME) and passed through a 40 μm cell strainer (Falcon) to remove cell aggregates. Five thousand GFP^+^mCherry^+^ pyrimethamine-resistant parasites were sorted by FACS (Moflo Astrios EQ, Beckman Coulter at FACS lab University of Bern, Switzerland: 25psi, 100 μm nozzle, 488nm and 561nm Lasers, 620/29nm and 526/52nm Filters), collected in 500 μL WME with 10% fetal bovine serum. 1,000 parasites were immediately injected intravenously into a naive mouse.

##### Genotyping of gene-deletion mutants

For the 3xHA-hdhfr-yFCU parasite lines, correct integration of the constructs and deletion of the genes were verified by Southern analyses of Pulsed Field Gel (PFG)-separated chromosomes and diagnostic PCR analysis (Janse et al., 2006). To show integration of the *Plasmo*GEM constructs containing h*dhfr::yfcu* (SM), the PFG-separated chromosomes were hybridized with a mixture of two probes: a probe of the h*dhfr* gene and a ∼800cbp fragment of the 5′UTR of PBANKA_0508000 located on chromosome 5 (Salman et al., 2015) for lines ΔFabH and ΔCBR or hybridized with a probe recognizing the 3′-UTR of *pbdhfr* (Salman et al., 2015) for all other lines. PCR primers used to confirm correct integration of the constructs are listed in Table S7.

Correct integration for GOMO-GFP-Cherry-FACS-based parasite lines and confirmation of the absence of targeted genes in all single knockout lines was confirmed by diagnostic PCR (Figures S4, S5, and S6). Primers from the PBANKA_0514900 gene (P28) were used to confirm the presence of gDNA for all single knockout lines (Figure S6) used for PCRs.

##### Genetic crossing of GOMO parasites

Genetic crosses were performed by infecting mice with equal parasite numbers of both WT (cl15cy1) and GOMO-GFP-mCherry-FACS parasite lines (ΔKCR_v2_, ΔPMM_v2_, ΔUAP) and allowing female mosquitoes to feed directly on these mice (Ecker et al., 2008, Rathnapala et al., 2017).

#### Phenotypic analysis of single knockout parasite lines

##### Blood stage phenotyping

To assess relative development of blood stage parasitemia, 100,000 asexual blood stage parasites (ΔKCR_V1_ or 1868cl1) were intravenously injected into each of three naive mice. The parasite growth was routinely recorded from day 1 until 6 post-injection by FACS (ACEA Novocyte machine).

##### Gametocyte production and ookinete formation assays

Gametocyte production of those lines showing reduced oocyst numbers was determined as described (Janse et al., 1985). The gametocyte conversion rate is defined as the percentage of ring-forms that develop into gametocytes in standard synchronized *in vivo* infections in mice. Ookinete formation assays were performed following published methods using gametocyte-enriched blood collected from mice treated with phenylhydrazine/NaCl (Beetsma et al., 1998). Briefly, infected blood containing gametocytes was mixed in standard ookinete culture medium in 24-well plates and cultures were incubated for 18-24 h at 21-22°C. Between 12-20 min, after activation of gametocytes, the number of exflagellating male gametocytes was determined by counting exflagellating males in a Bürker cell chamber. The fertilization rate (ookinete conversion rate), defined as the percentage of female gametes that develop into zygotes or ookinetes, was determined by counting female gametes and zygotes/ookinetes in Giemsa-stained blood smears at 18-24 h after *in vitro* induction of gamete formation. The different developmental stages (zygotes, developing ookinetes and mature ookinetes) were counted in Giemsa-stained slides according to the classification in Janse et al., 1985.

##### Midgut oocyst counting

For mosquito transmission experiments, 100-150 *A. stephensi* mosquitoes were allowed to feed for 1 h on anaesthetized infected mice whose asexual parasitemia had reached at least 7% determined by FACS and were carrying comparable numbers of gametocytes as determined on Giemsa stained blood films. Day 6-8 post feeding 10 mosquitoes were dissected in PBS1X and oocysts on their midguts counted. Images were recorded using a 5 × objective on a Leica DM 6000 B microscope fitted with a Leica DFC 350 FX camera. Pictures of the 10 mosquito midguts were taken and the oocyst number per midgut was recorded by using the software ImageJ (FIJI) for the 1868cl1 and the mutant parasite lines. Red or green fluorescent oocysts were recorded for the 3xHA-hdhfr-yFCU or GOMO-GFP-Cherry-FACS parasite lines respectively. The results were statistically evaluated using Prism (GraphPad) with a one-way analysis of variance (ANOVA) test with Dunnet’s multiple comparisons. At day 18-22 post feeding, salivary glands were dissected from up to 50 blood-fed infected mosquitoes, homogenized and assessed for the presence or absence of sporozoites.

##### *In vivo* analysis of liver stage phenotype

To analyze *in vivo* liver stage parasite development, at Day 18-22 post feeding, 3-15 mosquitoes were dissected and their salivary glands crushed with a homogenizer to release sporozoites, which were then quantified using a hemocytometer. Five thousand salivary gland sporozoites were intravenously injected into three female C57BL/6JRj (6weeks old, from Janvier) for the 1868cl1 or the mutant parasite line for each experiment. For the crossed parasites, sporozoites were counted using a 20x objective with green fluorescence to discriminate between GFP+mCherry+ mutant and cl15cy1 sporozoites and six rather than three mice were injected. Three of the mice injected with the crossed parasites were supplied with pyrimethamine pressure the day after injection to eliminate cl15cy1 parasites within the blood.

For ΔPDH-E2, ΔKCR (xWT), ΔCBR, ΔUSP, ΔPMM (xWT) and ΔUAP (xWT) parasites, parasitemia was monitored daily by FACS analysis of mouse tail blood (based on mCherry fluorescence for standard KO experiments and based on mCherry^+^GFP^+^ parasites for crossed knockout parasites. For ΔHCS1, ΔFabD, ΔFabG, ΔFabH, ΔLipA and ΔELO-A, relative blood stage parasitemia was determined by luciferase assay, taking advantage of the constitutive luciferase expression of the background 1868cl1 parasite strain. For this, 2 μl tail blood was added to 20 μl 1x Cell Culture Lysis reagent (Promega). Following incubation on ice for 1 min and vortexing for 10-15 s, the sample was centrifuged at 12,000 rpm for 15 s and the supernatant was stored at −20°C. 20 μl of this lysate was mixed with 100 μl Luciferase Assay Reagent (Promega) and relative light units were measured immediately using a Spectramax L Microplate Reader (Molecular Devices). A sample of uninfected blood was used as a control and relative light units for this sample were subtracted from those of experimental samples. In all cases, mice were allowed to reach a parasitemia of 2%.

##### *In vitro* analysis of liver stage phenotype

HeLa cells were seeded in a 96-well plate at high density the day before infection and were infected with ∼20,000 *P. berghei* parasites for 2 h. Infections were performed in triplicate. The infected cells were then detached using accutase (Innovative Cell Technology) and seeded a lower density. At 48 hpi, cells of one plate were fixed with 4%PFA/PBS for 10 min. Parasite size and number were determined by automated microscopy (IN Cell analyzer, GE lifesciences; Microscopy Imaging Center, University of Bern, Switzerland) monitoring the mCherry signal in the case of KO parasites generated with 3xHA-hdhfr-yFCU-based vectors and GFP in the case of KO parasites generated with GOMO-GFP-Cherry-FACS-based vectors. At 65 hpi the detached cells in the medium of infected cells were quantified by fluorescence microscopy (Olympus CKX41). The detached cell formation rate was calculated as the percentage of parasites at 48 hpi that form detached cells. The results were statistically evaluated using Prism (GraphPad) with a one-way analysis of variance (ANOVA) test with Dunnet’s multiple comparisons.

#### Generation of a *P. berghei* metabolic model

We generated the genome-scale metabolic model iPbe to integrate metabolic tasks (Table S3), genes, and the latest description of *Plasmodium* genes and metabolic reactions based on the PlasmoDB (Bahl et al., 2003), KEGG database (Kanehisa and Goto, 2000), ATLAS of Biochemistry (Hadadi et al., 2016), Malaria Parasites Methabolic Pathway (MPMP) database (Ginsburg and Tilley, 2011), and genome-scale model of *Escherichia coli* iJO1366 (Orth et al., 2011). We constructed iPbe with the RAVEN Toolbox (Agren et al., 2013) and curated iPbe thermodynamically within the matTFA toolbox (Salvy et al., 2019) (as done before for the genome-scale metabolic model of *P. falciparum* iPfa (Chiappino-Pepe et al., 2017a)) and following the newly generated PhenoMapping workflow. Here, we summarize the main steps followed in the reconstruction of iPbe. All references and details related to the previously available data and software used can be found in the key resources table.

##### Generation of a draft metabolic network

A draft metabolic network of an organism involves a set of enzymes (and its associated reactions and metabolites) based on the functional metabolic annotation of the organism’s genome. One could annotate the metabolic functions to the genome of an organism of interest using various sources of information: available experimental evidence, annotated genomes of closely related organisms, or blasting against previously characterized proteins, which are proteins with an annotated metabolic function.

We generated two draft metabolic networks of iPbe; a draft network that we annotated based on iPfa (with strictness value 2 and the default values of the parameters defined in the getModelFromHomology function in RAVEN), and a draft network that we newly annotated based on KEGG (with cut-off value of 10^−15^, minimal score ratio of 0.8, and minimal score ratio for a knocked-out gene of 0.3 as input for the function getKEGGModelForOrganism in RAVEN). We then merged the two draft metabolic networks to incorporate all annotated metabolic functions, using as reference the network obtained from iPfa. We included 63 genes that had been annotated based on KEGG and were not part of the draft metabolic network generated from iPfa.

##### Compartmentalization of the metabolic network or localization of enzymes

Eukaryotic cells have intracellular compartments where its proteins are localized. The localization of proteins in an organism might be determined through experimental localization, localization of proteins in closely related organisms, or identification of signal peptides in the protein sequence.

We assumed the localization of proteins in *P. berghei* is the same as in *P. falciparum*. Hence, we kept the localization from iPfa for all orthologous proteins. We localized the enzymes of the 63 new genes based on localization scores from TargetP (Emanuelsson et al., 2007), MitoProtII (Claros, 1995), and ApicoAP (Cilingir et al., 2012) software.

##### Definition of molecules required for growth or biomass building blocks

Genome-scale models define molecules that are cellular components or biomass building blocks. The production of all biomass building blocks is required for *in silico* growth.

We updated the biomass definition of iPfa by substituting ten biomass building blocks with twenty-one new downstream molecules based on the PhenoMapping analyses (see PhenoMapping workflow). Removed building blocks are: C00043_c (UDP-N-acetyl-D-glucosamine), C00096_c (GDP-mannose), C00143_c (5,10-Methylenetetrahydrofolate), C00325_c (GDP-L-fucose), C16237_a (Protein N6- (lipoyl)lysine), C17569_m (Ubiquinone-8), C00550_c (Sphingomyelin), C00120_c (Biotin), C00029_c (UDP-glucose), C00052_c (UDP-D-galactose). Added building blocks are: C00004_c (NADH), C00005_c (NADPH), C00415_c (Dihydrofolate), C04549_c (1-Phosphatidyl-1D-myo-inositol 3-phosphate), C19085_c (tRNA with a 3 CCA end), C04419_a (Carboxybiotin-carboxyl-carrier protein), G13044_r (Glycosylphosphatidylinositol (GPI)-anchor protein), G13052_r (Fucosylated protein), C00143_m (5,10-Methylenetetrahydrofolate), C15672_m (Heme O), C00126_m (Ferrocytochrome *c*), C20120_c (S-Farnesyl protein), C00550_r (Sphingomyelin), G00009_r (N-Glycan), C00344_r (Phosphatidylglycerol), quinone_m (Quinone), 2Fe2S_a ([2Fe-2S] iron-sulfur cluster), 2Fe2S_m ([2Fe-2S] iron-sulfur cluster), 4Fe4S_a ([4Fe-4S] iron-sulfur cluster), 4Fe4S_m ([4Fe-4S] iron-sulfur cluster), C00268_c (Dihydrobiopterin). We recalculated the stoichiometric requirements for the biomass building blocks following the procedure described for iPfa (detailed calculations in Table S3). Overall, iPbe includes 84 biomass building blocks (Table S3).

##### Definition of metabolite transportability

It is highly unknown what metabolites the malaria parasites can take up, secrete and transport inside the cell. In the absence of a specific transport mechanism, metabolites might diffuse through cell membranes. However, not all metabolites can be easily transported through cell membranes.

As done before in iPfa, we allow the transport of all cytosolic substrates that do not contain a phosphate, acyl-carrier protein, or CoA group. We also prevent the transport of big molecules like proteins. The medium of iPbe comprises 248 substrates that iPbe can take up and secrete, including substrates that the parasite shares with its host cells (erythrocyte and hepatocyte) based on previous mice genome-scale models (Sigurdsson et al., 2010) and metabolomics studies (Desai, 2013, Krebs, 1950). We followed the same transportability principles to allow the transport of metabolites between compartments within the parasite itself (Table S4).

##### Metabolic tasks and gap-filling

It is normal that draft metabolic networks cannot produce all biomass building blocks from the available substrates. This happens because we have an incomplete annotation of genomes. One should integrate into the draft network a set of hypothetical reactions also called gap-filling reactions to properly connect the metabolites within the metabolic network and allow the production of biomass. One can look for gap-filling reactions in available databases. The hypothetical metabolic capabilities in genome-scale models might be later characterized experimentally.

We first integrated into iPbe reactions that allowed the production of biomass building blocks using iPfa as a reference. Next, we gap-filled the network to achieve a set of metabolic tasks associated or not with growth (Table S3). We used the genome-scale model of *Escherichia coli* iJO1366 to identify reactions that produce iron-sulfur clusters. We used the KEGG database and the ATLAS of Biochemistry to integrate reactions that produce proteins, glycan, and linolate. We also defined manually the synthesis of a generic protein from the Aminoacyls-tRNA; we assumed the generic protein is formed of 200 amino acids based, which is the average protein length defined in the first sequence of *P. falciparum* genome (Gardner et al., 2002). The generic protein served as a substrate to produce biomass precursors containing proteins and allowed connecting the Aminoacyls-tRNA to the biomass production. We used the MPMP database and PlasmoDB to verify that all known metabolic capabilities and metabolic genes in the malaria parasite are part of iPbe. We mapped 67 genes to the gap-filling reactions based on the putative annotations suggested in PlasmoDB. These genes are marked in iPbe with the tag “putative_” before the gene ID and might require reannotation or validation in the future.

##### PhenoMapping to curate the metabolic network

Metabolic models should allow the generation of qualitative and quantitative predictions of the metabolic function. Despite the efforts in constructing high-quality metabolic models (Thiele and Palsson, 2010), the results from their computational analysis do not always match with the available data. Semi-automatic and iterative curation processes are needed to reduce the knowledge gaps and thereby increase the agreement between *in silico* and experimental observations. The curation process of a metabolic model can provide valuable insights into the metabolic processes and the biochemistry of an organism that are partially characterized in the literature and the metabolic model.

**Figure S8:**
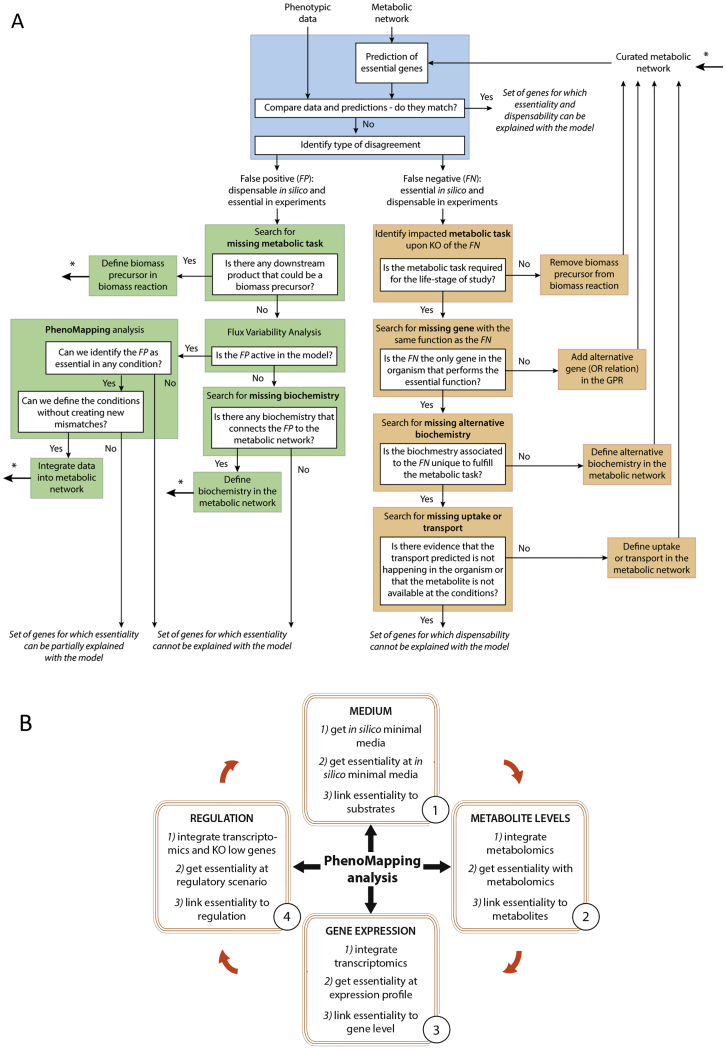
The PhenoMapping Workflow to Curate Metabolic Models and PhenoMapping Analyses to Identify Context-Specific Conditions Underlying Phenotypes, Related to Figure 4 (A) The PhenoMapping workflow involves three sets of analyses: (i) essentiality studies using the metabolic model and comparison with phenotypic data (blue); (ii) correction of False Negatives (FN, orange); and correction of False Positives (FP, blue). The curation of the metabolic network provides hypotheses on the metabolic function, here essential genes and underlying processes responsible for the essentiality. The classification of genes as fully, partially, or not agreeing with observed phenotypes might change when one modifies the metabolic network (at each iteration through the workflow). GPR denotes the gene-protein-reaction associations in the metabolic model. (B) PhenoMapping analysis to study four context-specific cellular processes or conditions underlying phenotypes: the uptake of substrates, thermodynamic directionality of reactions and transports for a set of context-specific metabolite concentrations, gene expression, and regulation of gene expression. The PhenoMapping analysis is modular: linear arrows from the center of the figure to the boxes of cellular processes indicate an independent PhenoMapping analysis of cellular processes; and curved arrows between cellular processes indicate that the PhenoMapping analysis can be applied cumulatively following the order number suggested.

We applied the PhenoMapping workflow (Figure S8, description in PhenoMapping workflow section) to redefine metabolic tasks (biomass building blocks) and gene-protein-reaction associations, and integrate new alternative biochemistry and uptakes or secretions. We used blood- (Bushell et al., 2017) and liver- (this study) stage specific growth phenotypes to refine the reconstructed metabolic network and avoid the prediction of essential genes that are observed as dispensable in any of the life stages (false negatives, Table S3). In this way, we generated a generic and life-stage agnostic metabolic network of *P. berghei* that we call iPbe.

##### Thermodynamic curation of iPbe

The directionality of reactions in a metabolic network determines what metabolic pathways can be used for growth. The thermodynamic curation of a genome-scale model enables the calculation of the thermodynamically feasible directionality of its metabolic reactions at some experimentally determined intracellular conditions.

We integrated into iPbe available experimental information of the intracellular conditions in *P. berghei*, namely pH, ionic strength, membrane potential of intracellular compartments (primarily obtained from (Mohring et al., 2017), see Table S3), and intracellular concentration ranges of metabolites. We calculated the thermodynamic properties of metabolites and reactions in iPbe and integrated them in the form of thermodynamic constraints following the systematic approach defined within the framework of thermodynamics-based flux analysis (TFA) in the matTFA toolbox. We generated two thermodynamically curated versions of iPbe: one for the analysis of the blood stages (iPbe-for-blood) and one for the analysis of the liver stages (iPbe-for-liver). These versions differ in the pH value of the parasitophorous vacuole, the upper bound of the extracellular (outside the parasite) metabolite concentration, and the possibility of taking up oxyhemoglobin only in the blood stages. We assumed that the pH of the parasitophorous vacuole in the liver stages was the same as the pH of the hepatocyte’s cytosol (Table S3). In both versions, we allowed the concentration of every intracellular metabolite to vary between 1 μM and 50 mM, which covers the intracellular concentrations of a wide range of metabolites in various cells and conditions (Bennett et al., 2009, Ishii et al., 2007, Teng et al., 2009, Teng et al., 2014, Vo Duy et al., 2012). We assumed that the concentrations of metabolites outside the parasite (host cell and blood serum) are more flexible to vary than inside the parasite cell. In this way, the concentrations of metabolites outside the parasite cell were allowed to vary between 0.01 μM and 100 mM in the blood stages and between 0.01 μM and 80 mM in the liver stages.

#### PhenoMapping workflow

We propose the PhenoMapping workflow (Figure S8) to (i) generate highly curated non-context specific and context specific metabolic models, (ii) identify unconditional and conditional essentiality, and (iii) suggest mechanistic origin of phenotypes. This workflow integrates previously suggested concepts (Barua et al., 2010, Benedict et al., 2014, Hartleb et al., 2016, Kumar and Maranas, 2009, Satish Kumar et al., 2007, O’Brien et al., 2015, Sohn et al., 2012, Tervo and Reed, 2013), and new concepts and analysis developed in this study (see next subsections) into a semi-automatic framework. Inputs to this workflow are: a metabolic model and (at least) one phenotypic dataset. In this study, we used growth/non-growth phenotypes.

The workflow involves three sets of analyses (Figure S8). We perform essentiality studies using the metabolic model and compare the essentiality predictions with available phenotypic data. The comparison points out disagreements: False Negatives (FN) when there is growth *in vivo* but the model predicts no growth, and False Positives (FP) when there is no growth *in vivo* but the model predicts growth.

We first suggest a reconciliation of FNs to correct or add missing information in the model. We sequentially evaluate the redefinition of metabolic tasks and GPRs, and the integration of missing alternative biochemistry and uptakes or secretions into the metabolic model. We study the metabolic tasks impacted upon knockout of the FN gene. Such studies identify the biomass precursors whose production requires the function of the FN gene. One might identify a molecule whose production is only required in one context of study and hence should not be defined as a biomass precursor in a generic model. For example, in a previous version of iPbe, the requirement of lipoate production in the apicoplast organelle was responsible for the incorrect essentiality prediction of the fatty acid synthesis (FAS) II in the blood stages (Bushell et al., 2017, Shears et al., 2015). The FAS II in the malaria parasites is known to be essential in the liver stages but not in the blood stages (Shears et al., 2015). We eliminated the requirement of lipoate production in the apicoplast in iPbe to model the metabolism of the parasites. A lack of genes or reactions in the metabolic model is also responsible for the observation of FNs (Orth and Palsson, 2010, Thiele and Palsson, 2010). For example, we might identify *in silico* a gene as essential because the metabolic model lacks information regarding another protein carrying out the same function (an isoenzyme). Therefore, we looked for missing genes, intracellular reactions, or transports/uptakes/secretions in the metabolic model following a gap-filling approach. This information is missing in the metabolic model due to uncertainty in the functional annotation of the genes, the uncertainty in the transportability of metabolites across membranes, and the limited knowledge concerning the (lack of) specificity of enzymes. We can reconcile such cases through the integration of genes with higher uncertainty in the annotation. We integrated into iPbe 67 genes with putative annotation based on PlasmoDB that were not part of the initial iPbe.

Next, we suggest a reconciliation of FPs (green) to first add missing biomass precursors and then constraints that describe a context-specific behavior. If the FPs are not blocked based on a flux variability analysis, we perform a PhenoMapping analysis to identify the conditions in which the FPs become essential. If the FPs are blocked or inactive, we perform gap-filling to connect the FP genes and reactions to the metabolic network and send the model to a second iteration through the PhenoMapping workflow. For example, in iPbe, we defined the GPI-anchored proteins (downstream metabolite) instead of the nucleotide sugars (upstream metabolite) as biomass precursors to account for the essentiality of GPI-anchor biosynthesis. In addition, we performed gap-filling (i.e., definition of transporters for nucleotide sugars between cytosol and endoplasmic reticulum) to allow the production of the GPI-anchored proteins in the endoplasmic reticulum.

#### Essentiality prediction with iPbe

We sought for the essential genes and reactions for liver and blood development in iPbe-liver and iPbe-blood, respectively. We performed *in silico* analysis of essential genes and reactions on iPbe, iPbe-liver and iPbe-blood using the Fast-SL approach (Pratapa et al., 2015) within FBA (Varma and Palsson, 1994, Orth et al., 2010) in the COBRA (Heirendt et al., 2007, Schellenberger et al., 2011) and matTFA (Salvy et al., 2019, Henry et al., 2006, Henry et al., 2007) Toolbox. In the Fast-SL approach, a set of genes and reactions that are not required for optimal growth (here defined as the objective function) based on a parsimonious FBA, pFBA (Lewis et al., 2010) are discarded. The remaining set of genes or reactions are exhaustively knocked out and growth is tested. We defined three classes of *in silico* phenotypes based on the ranges of relative growth (KO/WT) predicted: between (1 and 0.9); (0.9 and 0.1); and (0.1 and 0) determined a dispensable, slow and essential *in silico* phenotype, respectively. The predictions of essential genes with iPbe, iPbe-blood and iPbe-liver are available in Table S3.

#### Evaluation of gene essentiality predictions

The essentiality predictions in iPbe-liver with TFA were compared with the liver phenotypes obtained in this study. The comparison of *in vitro* and *in vivo* observations provided a measure of the genome-scale model’s accuracy to describe the metabolic function at the life stage in which the phenotypes were obtained, i.e., liver stages of the *Plasmodium* infection. The accuracy score was calculated as (TP+TN)/(TP+TN+FP+FN), where TP (true positive) and TN (true negative) define predictions that correctly simulate growth and non-growth, respectively, based on the available experimental data. While FP (false positive) and FN (false negative) describe predictions that incorrectly simulate growth and non-growth, respectively. We also calculated the Matthew Correlation Coefficient (MCC) as (TP x TN – FP x FN)/((TP+FP) x (TP+FN) x (TN+FP) x (TN+FN))^0.5^. In iPbe-liver, an accuracy score of 85% and a MCC of 0.51 are obtained based on the liver phenotypes of this study (STAR Methods). *Mild* liver phenotypes (2-100 fold reduction) and genes blocked in iPbe (the metabolic model before integration of context-specific data) were not considered in the calculation of the accuracy score and MCC. Moreover, we evaluated the predictions per metabolic subsystem with the liver phenotypes from this study. We calculated a measure for agreement per metabolic subsystem as (TP+TN+0.5^∗^MP)_subsystem i_ / (FP+FN+TP+TN+MP)_subsystem i_, where MP defines the number of *mild* phenotypes that are part of the subsystem. We called this measure degree of agreement (DoA) and the results are presented in Figure 4B. The analysis was done for all genes with a liver phenotype. Overall, there are liver phenotypes available for 157 genes out of the total 428 genes in iPbe-liver (Table S3).

#### PhenoMapping analysis

##### Classification of information/cellular processes integrated into a genome-scale model

Genome-scale models are powerful platforms for the study of metabolism. Predictions on the metabolic function of an organism obtained from a genome-scale model are the product of some biological information or assumptions integrated into the model like the association of genes and enzymes, biochemistry defined in the metabolic network, or other types of data specified in the model in the form of constraints. A classification of the information integrated into a genome-scale model and mapping of this information to cellular processes will help us understand what cellular processes underlie predicted phenotypes.

We classify the information integrated into a genome-scale model in *organism-specific* and *context-specific* information (Figure 4) and map this information to a set of cellular processes. Organism-specific information refers here to static characteristics in metabolic models that do not vary with the context, condition, or life-stage. For example, we define as organism-specific information the metabolic functions annotated to the genome of the organism, the irreversibility and localization of the metabolic enzymes, and the transportability of metabolites across cellular membranes. Such information represents in metabolic models a static scaffold for further integration of context-specific data in the form of constraints. Context-specific information refers to the metabolic characteristics that are specific to a context/condition/life-stage of study. For example, we define as context-specific information the availability of a set of substrates, the possibility of metabolite secretion, the metabolite concentrations and associated thermodynamic feasibility of metabolic reactions and transports, the gene expression, and the regulation of gene expression. Genome-scale models integrate context-specific information to describe a cellular state. We describe below the details of the PhenoMapping analysis. All references and details on the data and software used can be found in the key resources table.

##### Classification of genes as unconditionally and conditionally essential

It is known that growing conditions/contexts/life-stages determine what genes are essential for growth in an organism. It is possible that a gene is essential in all growing conditions or it is only essential in a specific condition. Understanding what genes are essential for growth in all growing conditions and what genes arise as essential in specific conditions will help in the understanding of the metabolic function and design of drug targeting strategies.

We classify the essential genes and reactions into two types: unconditionally essential and conditionally essential. The classification is based on the type of information integrated into the metabolic model that leads to the essentiality. The static or organism-specific information of the model explains unconditional essentiality, since those genes are essential for a phenotype like growth in all contexts of study. We define a unique set of unconditionally essential genes when no context-specific data is integrated into the metabolic model of study. The context-specific information integrated into the metabolic model is responsible for the conditional essentiality, since those genes are only essential in some contexts of study. We identify conditional essential genes following the analyses described next.

##### Analysis of *in silico* minimal media (IMM)

Some organisms such as *Escherichia coli* and *Saccharomyces cerevisiae* can grow on glucose and some inorganics as the only extracellular compounds. However, parasites such as *Plasmodium berghei* rely on the uptake of additional compounds. The dependency of the malaria parasites on the host cell (regarding nutritional requirements) is not yet fully understood. The aim of the analysis of *in silico* minimal media (IMM) is to identify the minimum number of substrates that the cell needs for growth and if applicable the number of alternative sets of IMMs.

As applied before in iPfa (Chiappino-Pepe et al., 2017a), the IMM algorithm identifies the minimal number of substrates that the cell needs for a cellular phenotype, such as biomass production. The IMM involves a Mixed Integer Linear Programming (MILP) formulation (optimization problem, OP, 1),

$$\min\sum_{i=1}^{M}y_{i}$$

$$s.t.S_{ij}\cdot v_{j}=0_{i},v_{min,j}\leq v_{j}\leq v_{max,j}$$

$$v_{j}=v_{f,j}-v_{r,j},v_{f,j},v_{r,j}\geq 0$$

(OP 1) $$v_{min,biomass}=p_{OGR}\cdot v_{opt,biomass}$$

$$v_{r,i}+F_{max}\cdot y_{i}\leq F_{max}$$

$$v_{r,i}+y_{i}\geq F_{min}$$

$$y_{i}\in \left\{0,1\right\}$$

where *M* is the total set of transport reactions between the extracellular environment and cytosol. Here, the net flux through a transport reaction *i* is given by the exclusive use of its forward flux *v*_*f,i*_ (secretion) or its reverse flux *v*_*r,i*_ (uptake), which are always positive. In the OP 1, an integer variable *y*_*i*_ indicates whether an uptake reaction *v*_*r,i*_ within the set of transport reactions *M* is inactive (*y*_*i*_ = 1) or active (*y*_*i*_ = 0). An uptake reaction *i* is inactive (*y*_*i*_ = 1) when the reverse flux *v*_*r*_ is blocked (*v*_*r,i*_ = 0). Otherwise, an uptake reaction *i* is active (*y*_*i*_ = 0) when it carries a minimal flux *F*_*min*_, which is defined here as 10^−7^ mmol/g-DW/h (*v*_*r,i*_ > 10^−7^). We also define a capacity constraint with the maximal flux that a reaction can carry *F*_*max*_, which is 50 mmol/g-DW/h. The resolution of the OP 1 identifies the maximum number of uptake reactions that can be blocked and allow a minimum growth requirement. We defined the growth requirement by setting the lower bound of the biomass function at a 10%, *p*_*OGR*_, of the optimal growth yield, *v*_*opt,biomass*_. The uptake reactions that are active after the optimization of the OP 1 constitute an IMM. We identify alternative IMMs by integrating integer cut constraints after generating each solution iteratively (Lee et al., 2000) (Equation 1),

(eq. 1) $$\sum_{k}^{M}y_{M_{k}}>0$$

where *M* is the set of transport reactions between the extracellular environment and cytosol (same as OP 1). The set *M*_*k*_ is the subset of the transport reactions in *M* that have inactive integers (*y*_*M,k*_ = 0) in a solution *k* of the OP 1. The cut constraint enforces that the next solution *k+1* has at least one integer of the set *M*_*k*_ different.

##### Analysis of *in silico* minimal secretion (IMS)

All cells secrete metabolites that are byproducts of growth and maintenance of the cell function. In parasites like *Plasmodium berghei* the secretion of such metabolites might affect the metabolism of the host cell. It is unknown what metabolites the malaria parasites need to secrete during infection in the blood and liver stages. The aim of the analysis of *in silico* minimal secretion (IMS) is to identify the minimum number of substrates that the cell needs to secrete to sustain a phenotype like growth and if applicable the number of alternative sets of IMSs.

An analogous OP to OP 1 allows the study of IMS. In an IMS formulation the integer variable of a reaction *i* controls its forward flux *v*_*f,i*_ instead of its backward flux *v*_*r,i*_.

##### Analysis of essential genes per IMM and IMS

The composition of the extracellular medium might have an effect on gene essentiality. The analysis of essential genes per IMM and IMS aims to identify what genes can become essential due to substrate inaccessibility and due to impossibility of metabolite secretion, respectively.

As done before for iPfa (Chiappino-Pepe et al., 2017a), we define each IMM in iPbe and we perform gene essentiality at each IMM. We then identify genes that are essential at the IMMs and not at the *in silico* rich medium of 248 substrates. Analogously, we also define the IMSs in iPbe and perform gene essentiality at each IMS. We identify the genes that are essential in the IMSs and not in the *in silico* rich medium.

##### Identification of bottleneck substrates or secretions

The analysis of bottleneck substrates finds the substrates that one should add to an IMM to render non-essential a gene that is essential in such IMM. This method requires the prior identification of all alternative IMM compositions and the essential genes in the IMMs, as explained above. For an IMM and upon knockout of a gene that is essential in this IMM and not in the rich medium, the analysis of bottleneck substrates identifies alternative sets of substrates to add to the IMM and rescue the phenotype, in this case growth. This method uses a mixed-integer linear programming (MILP) formulation (OP 1). We apply the OP 1 when the gene of study is knocked out and when the composition of the IMM at which the gene becomes essential is excluded from the set *M* of uptake reactions. The exclusion of the IMM substrates from the set *M* of uptake reactions assures that the IMM substrates are available to the cell, and enables the identification of a set of substrates that one should add to the IMM to rescue growth. We identify alternative sets of substrates by integrating integer cut constraints (Equation 1) after generating each solution iteratively. We follow the same approach to identify secretions responsible for gene essentiality in the IMS formulation.

##### Integration of metabolomics data

Intracellular metabolite concentrations have an effect on the thermodynamic properties of the metabolic reactions and reaction directionality. The logarithm (*ln*) of the metabolite concentration (mol/L_cell_) ranges were integrated within the TFA framework. The lowest and highest values correspond to the lower and upper bounds of the concentration variable in TFA, respectively. The constrained range of concentration limits the allowable *Δ*_*r*_*G’* range of the reactions in which the metabolites participate and with it the flux ranges of the neighboring reactions. The physiological or generic range of concentration of 1 μM and 50 mM was considered for a metabolite if no data was available (Teng et al., 2009, Teng et al., 2014, Vo Duy et al., 2012). For the metabolites present in more than one intracellular compartment, the same concentration range was defined in all of these compartments. Upon integration of metabolomics data, we performed a standard TFA to account for the new metabolomic constraints.

##### Identification of bottleneck metabolites

Genes and reactions might become essential in a metabolic model when we account for thermodynamic constraints and metabolomics data. In such cases, we consider that a thermodynamic bottleneck originates the essentiality of the gene. The goal of the analysis of bottleneck metabolites is to identify the metabolites whose concentrations or concentration ratios are responsible for the essential function of a gene. The allowable concentration range of the bottleneck metabolites should increase to relax the directionality of a set of reactions (i.e., reactions that are unidirectional or blocked become bidirectional or unblocked), and thereby a gene that is essential becomes non-essential.

As presented before for iPfa (Chiappino-Pepe et al., 2017a), the analysis of bottleneck metabolites identifies the metabolites responsible for the thermodynamic bottlenecks. Bottleneck metabolites determine the directionality of a set of reactions and allow the identification of new essential genes. We knock out the new essential genes in iPbe one-by-one/separately. With these genes knocked out and metabolomics data integrated (Teng et al., 2009, Teng et al., 2014, Vo Duy et al., 2012), iPbe growth is feasible with FBA but not with TFA. An MILP formulation (OP 2) was defined to search for the minimal number of metabolites whose concentration ranges cannot be integrated or should be relaxed (increased) to make the model feasible in TFA,

$$\min\sum_{i=1}^{M}z_{i}\;$$

$$s.t.S_{ij}\cdot v_{j}=0_{i},v_{min,j}\leq v_{j}\leq v_{max,j}$$

s.t.(TFAconstraints)

(OP 2) $$v_{min,biomass}=p_{OGR}\cdot v_{opt,biomass}$$

$$\ln C_{i}+\left(\ln C_{EXPmin,i}-\ln C_{min,i}\right)\cdot z_{i}>\ln C_{EXPmin,i}$$

$$\ln C_{i}+\left(\ln C_{EXPmax,i}-\ln C_{max,i}\right)\cdot z_{i}<\ln C_{EXPmax,i}$$

$$v_{KO}=0$$

$$z_{i}\in \left\{0,1\right\}$$

where an integer variable *z*_*i*_ is associated to each metabolite concentration range in the set *M* of metabolites for which experimental concentration data is available. The variable *z*_*i*_ is inactive (*z*_*i*_ = 0) when a metabolite *i* can keep its experimental concentration range, *C*_*i*_ ∈ (*C*_*EXPmin,i*_, *C*_*EXPmax,i*_). We define the experimental concentration range of a metabolite *i* with an upper concentration value, *C*_*EXPmax,i*_, and a lower concentration value, *C*_*EXPmin,i*_, based on the standard deviation from the average measurement reported in the metabolomics study. We work with concentration values in mol per unit of volume in liters of cell.

The variable *z*_*i*_ is active (*z*_*i*_ = 1) when the concentration range of a metabolite *i* should be relaxed (increased), *C*_*i*_ ∈ (*C*_*min,i*_, *C*_*max,i*_), compared with its experimental concentration range. In this way, the new minimal concentration *C*_*min,i*_ is lower than the minimal experimental concentration *C*_*EXPmin,i*_ (*C*_*min,i*_ < *C*_*EXPmin,i*_), and the new maximal concentration *C*_*max,i*_ is greater than the maximal experimental concentration *C*_*EXPmax,i*_ (*C*_*EXPmax,i*_ < *C*_*max,i*_).

The experimental (if *z*_*i*_ = 0) or the relaxed (if *z*_*i*_ = 1) concentration ranges determine the bounds of the logarithm (ln) of the metabolite concentration *C*_*i*_, which is a variable in the TFA framework. The logarithm of the metabolite concentration (ln(*C*_*i*_)) will serve to calculate the Gibbs free energy of the reaction (*Δ*_*r*_*G’*).

Here, a gene becomes essential when we account for thermodynamic constraints and some metabolite concentrations belong to the experimental concentration range *C*_*i*_ ∈ (*C*_*EXPmin,i*_, *C*_*EXPmax,i*_). We consider a gene is essential when its knockout leads to a reduction in optimal wild-type growth of 90% or more. Hence, we defined the growth requirement of the OP 2 by setting the lower bound of the biomass function at a 10%, *pOGR*, of the optimal growth yield, *v*_*opt,biomass*_. This analysis is done when the gene of study is knocked out, which implies that all its associated reactions are knocked out based on the gene-protein-reaction associations (*v*_*KO*_ = 0).

We identify alternative sets of metabolites by integrating integer cut constraints after generating each solution iteratively (Lee et al., 2000) (Equation 2). We redefine the integer cuts defined for the maximization problem (Equation 1) to avoid obtaining suboptimal solutions that are supersets (in terms of integers) of an optimal solution.

(eq. 2) $$\sum_{k}^{M}\left(1-z_{M_{k}}\right)>0$$

where *M* is the set of metabolites for which experimental concentration data is available (same as OP 2). The set *M*_*k*_ is the subset of the metabolites in *M* that have active integers (*z*_*M,k*_ = 1) in a solution *k* of the OP 2. The cut constraint enforces that the next solution *k+1* has at least one integer of the set *M*_*k*_ different.

##### Integration of gene expression data with TEX-FBA

Transcriptomics data (here RNaseq) quantify the amount of mRNA present in a cell at a certain time point of its life cycle, thereby suggesting which genes and reactions might be active at that time point. Gene expression might change between the blood and liver stages of *P. berghei* development. TEX-FBA is the only methodology that allows to integrate gene expression data into genome-scale models and account simultaneously for thermodynamics and metabolomics data to generate context-specific models.

The TEX-FBA methodology (Pandey et al., 2019) maximizes associations between levels of gene expression and levels of reaction fluxes. The inputs to TEX-FBA are a model, a set of lowly, medium, and highly expressed genes (based on absolute gene expression levels), and two flux thresholds *p*_*l,*_ and *p*_*h*_ to associate to lowly and highly expressed reactions. TEX-FBA translates gene levels to reaction levels using the gene-protein-reaction rules in the model. We assumed that the combination of lowly and highly expressed genes in a gene-protein-reaction rule with OR and AND associations allow a high and low flux, respectively, through the associated reactions. TEX-FBA then performs flux variability analysis (FVA) to identify the allowable flux ranges (FR) of the reactions at the conditions of study, and associates within the FVA ranges a maximum allowable flux through the lowly expressed reactions and a minimum required flux through the highly expressed reactions using a MILP formulation. For high-labeled reactions we enforced a minimal flux such that FR = [*p*_*h*_ *.(v*_*max*_*-v*_*min*_*), v*_*max*_], while we enforced a maximum flux value for low-labeled reactions such that FR = [*v*_*min*_*, p*_*l*_ *.(v*_*max*_*-v*_*min*_*)*]. The maximum number of highly and lowly expressed reactions that satisfy the associated FRs define a *maximum consistency score*. TEX-FBA allows the identification of all alternative gene expression profiles for the maximum consistency score. See next sections for a description of the input parameters in the integration of gene expression into iPbe.

##### Identification of essential genes in alternative gene expression profiles

Gene essentiality might change based on gene expression. For instance, cells might have redundant metabolic pathways to produce an essential metabolite. If both pathways are active simultaneously (their associated genes are expressed), they will be part of synthetic lethal pairs. If only one of these pathways is active, it will be essential for growth. The goal of this approach is to identify genes that become essential upon integration of gene expression data into a genome-scale model.

We integrated gene expression data following the TEX-FBA approach (Pandey et al., 2019), and we identified all alternative gene expression profiles at maximum consistency score. We defined separately each gene expression profile in the model by keeping the flux constraints on the highly and lowly expressed reactions and keeping the requirement of maximum consistency score. We then performed gene essentiality analysis following a standard exhaustive enumeration approach with TFA, i.e., knocking out the genes one-by-one and testing if growth could be achieved. Here, any gene whose knockout led to an infeasible solution was considered essential.

##### Identification of essential genes in a scenario without regulation of gene expression between isoenzymes

Transcriptional regulation links metabolism and gene expression in the cell (Chiappino-Pepe et al., 2017b, Donati et al., 2018). Upon knockout of a gene, its isoenzyme might become upregulated to compensate for this loss and assure the flux through the metabolic pathway. In this study, we considered the extreme scenario in which lowly-expressed genes are knocked out (there is no regulation), and we studied *in silico* essentiality with transcriptomics data integrated. In this context, a lack of regulation indicates that a lowly expressed gene cannot get upregulated to compensate for the knockout of a highly expressed gene.

We knocked out all lowly expressed genes by removing them from the GPR associations. We then identified essential genes at alternative gene expression profiles as described above.

##### Identification of bottleneck reaction levels

Integration of gene expression data into a genome-scale model rewires the distribution of fluxes in the metabolic network. New genes might become essential due to gene expression. One could identify reactions whose flux levels at a gene expression profile render a gene essential.

After identification of the gene expression profiles within TEX-FBA (Pandey et al., 2019) and the essential genes at the expression profiles, we identify alternative sets of lowly and highly expressed reactions whose expression constraints should be relaxed to rescue growth. This method uses a mixed-integer linear programming (MILP) formulation.

#### From context/condition/life-stage agnostic to specific models with PhenoMapping

PhenoMapping maps predicted phenotypes (here *in silico* essential genes for growth) to underlying cellular processes (here media composition, metabolite concentration levels, gene expression or regulation of gene expression). One can compare the predicted phenotypes with available experimental phenotypic data to suggest cellular processes that might happen in the conditions/context/life-stage studied. Such hypotheses on context-specific cellular physiology can guide the generation of context-specific metabolic models.

We compare the predicted phenotypes in all conditions studied with available experimental phenotypic data. When a gene is essential experimentally, and we identify it as essential with PhenoMapping, we consider keeping the underlying cellular process (constraint in the model). For example, there is a gene predicted as essential at an IMM and thanks to PhenoMapping we know the substrates that should be available at the IMM to make this gene non-essential. When the experimental phenotype describes this gene as essential, we can suggest the substrates mapped might not be available to the cell. Similarly, when the experimental phenotype defines this gene as dispensable, we hypothesize the substrates mapped might be available to the cell in the conditions studied. We note that there are genes with different experimental phenotypes (essential versus dispensable) that are mapped to the same cellular process. In this case, we suggest treating as dispensable all genes associated to this cellular process, which will avoid false negatives (genes predicted as essential while they are dispensable). We suggest performing the PhenoMapping analysis with a context/condition/life-stage agnostic metabolic model that integrates thermodynamic constraints with generic concentration ranges. We generate a context-specific metabolic model by integrating information into this context-agnostic model in the following order: we first define the available substrates, we second constraint the possibility of secretion, we third integrate metabolomics data, we next integrate transcriptomics data. Any of these steps might be skipped if there is no omics data available, e.g., we did not integrate metabolomics data into iPbe-blood or iPbe-liver.

We note that the final context-specific model that integrates simultaneously more than one cellular process might present new *in silico* essential genes that we did not identify in the independent analysis of cellular processes with PhenoMapping. PhenoMapping could also be applied to perform combinatorial studies of cellular processes that are simultaneously responsible for a phenotype. A combinatorial analysis was not performed here.

#### Generation of the blood- and liver-stage specific *P. berghei* metabolic models

We provide here details of the PhenoMapping analysis on iPbe used to generate iPbe-blood and iPbe-liver, to be read in parallel with Table S3 and with the end metabolic models and result of the pathways/genes shown in Table S4. All references related to the data and software used can be found in the key resources table.

##### Classification of genes as unconditionally and conditionally essential in iPbe

To find *in silico* unconditionally essential genes in iPbe, we performed essentiality studies with TFA using a rich medium with 248 substrates, and without integrating metabolomics or transcriptomics data into iPbe. We identified 118 such unconditionally essential genes in iPbe (Table S3, tab S3.2, column “iPbe essential genes (118)”). The accuracy of iPbe to predict blood- and liver-stage phenotypes is 0.72 and 0.77, with Matthew Correlation Coefficient of 0.53 and −0.06, respectively. The conditionally essential genes are identified in the analyses defined below.

##### Analysis of IMM in iPbe

The rich medium in iPbe comprises 248 substrates (see definition of media in reconstruction of iPbe). We searched for the *in silico* minimal medium (IMM) (Chiappino-Pepe et al., 2017a) (method detailed above) that allows growth in iPbe when the uptake of oxyhemoglobin is (iPbe-for-blood) and is not (iPbe-for-liver) allowed. The minimum number of substrates required for growth was 18 and 27, respectively. We found 144 and 2344 alternative compositions of IMM in iPbe for the two medium compositions (Table S3).

##### Analysis of IMS in iPbe

We looked for *in silico* minimal secretion (IMS) sets that allow growth in iPbe under all the 248 possible secretion conditions. The IMS results are the same in iPbe-for-blood and iPbe-for-liver. There is at least one metabolite that should be secreted when growth is required and there are 11 alternative such metabolites (Table S3).

##### Analysis of essential genes per IMM and IMS in iPbe

We used iPbe and the alternative IMM compositions to identify genes that become essential upon substrate inaccessibility. For each alternative IMM, we performed essentiality analysis, and we identified a total of 89 and 99 genes that became essential in the IMMs in iPbe-for-blood and iPbe-for-liver. The 89 and 99 genes are part of a group of genes whose essentiality depends on the conditions of study, also referred here as conditionally essential genes (Table S3).

Analogously, we performed essentiality with each IMS composition. There is a total of 43 genes predicted as essential at minimal secretion conditions.

##### Identification of bottleneck substrates or secretions in iPbe

We looked for substrates whose absence in the IMM and IMS is responsible for the essentiality of the conditionally essential genes, also defined as *bottleneck substrates* and *bottleneck secretions*. We found alternative sets of bottleneck substrates and secretions linked to the medium-related conditionally essential genes (Table S3). For example, the amino sugar GDP-mannose and myo-inositol are bottleneck substrates, whose absence from the medium leads to essentiality in the metabolic pathway of sugar activation (5.4.2.8/PBANKA_0501700 and 2.7.7.13/PBANKA_1022300) and the hydrolysis of inositol 3-phosphate (3.1.3.25/PBANKA_0803200), respectively (Table S3).

##### Integration of metabolomics data into iPbe and essentiality studies (test case)

We integrated metabolomics data within the TFA framework into iPbe-for-blood and iPbe-for-liver. We used a metabolomics dataset previously measured (Teng et al., 2009, Teng et al., 2014, Vo Duy et al., 2012) and compiled in the analysis of iPfa (Chiappino-Pepe et al., 2017a), which includes metabolite levels for 60 unique metabolites (affecting 142 compartmentalized metabolites) in iPbe. We found eight essential genes in iPbe-for-blood and two in iPbe-for-liver when this metabolomics dataset is integrated (Table S3).

##### Identification of bottleneck metabolites in iPbe (test case)

As defined before (Chiappino-Pepe et al., 2017a), bottleneck metabolites are those whose concentration ranges determine the directionality of a set of reactions and with it render genes essential for growth. We searched for bottleneck metabolites within the metabolomics dataset using iPbe-for-blood and iPbe-for-liver. There are nine bottleneck metabolites that involve important coenzymes, such as ATP and NAD+, and also nucleotides, and nucleotide sugars (Table S3). Interestingly, although the metabolomics data were obtained from *P. falciparum*, four out of eight blood-stage conditionally essential genes linked to metabolite levels show slow phenotypes in the blood stages of *P. berghei*. The only blood-stage dispensable gene in the set of eight metabolomics-related conditionally essential genes is the glucose phosphate transferase enzyme in the amino sugar metabolism (2.7.7.64, PBANKA_1232300). The analysis of bottleneck metabolites suggests that the concentration ratio between UDP-glucose and UMP in the cytosol is responsible for the essential function of PBANKA_1232300. When we disregard the experimental concentration range of UDP-glucose and allow its concentration to vary between 1 μM and 50 mM, the gene PBANKA_1232300 becomes dispensable in iPbe. Overall, the integration of metabolomics data from *P. falciparum* into iPbe increases the consistency with the blood-stage phenotypes but not with the liver-stage phenotypes. These results might suggest that the intraerythrocytic *P. berghei* shows similar metabolite levels to those measured in blood stage trophozoites of *P. falciparum*. Despite this observation, we did not integrate metabolomics data into iPbe.

##### Integration of gene expression data with TEX-FBA into iPbe

We integrated transcriptomics data (RNaseq for the blood and liver stages (Otto et al., 2010, Caldelari et al., 2019)) into iPbe using TEX-FBA. We selected the time points of maximum metabolic activity in both stages: 24 h and 48 h for the blood and liver stages. We defined the following parameters within TEX-FBA in all scenarios *p*_*l*_ = 2x10^−5^, *p*_*h*_ = 2 x10^−3^, low P value = 25, and high P value = 75. There is a unique blood-stage and two liver-stage specific metabolic profiles that renders a maximum consistency score.

##### Identification of essential genes with alternative gene expression profiles in iPbe

We identify 64 blood- and 70 liver-stage specific essential genes, with 47 genes that are essential in both life stages based on gene expression data. Out of the 64 genes that are *in silico* blood-stage essential, there are blood phenotypes for 49 genes and these define 24 essential genes, 7 slow genes, and 18 dispensable genes. Similarly, out of the 70 genes that are *in silico* liver-stage essential, there are liver phenotypes for 34 genes and these define 8 essential genes, 7 slow genes, and 19 dispensable genes (Table S3). Interestingly, the genes that show essential blood phenotypes participate in pathways of the central carbon metabolism, such as glycolysis and pentose phosphate pathway. Intraerythrocytic malaria parasites largely rely on a high glucose uptake to survive (Divo et al., 1985, Homewood, 1977). Glycolysis in the malaria parasites has hence been suggested as a drug target (Harris et al., 2013, Roth, 1990). Our PhenoMapping analysis suggests that the essential function of glycolysis in *P. berghei* is linked to the trophozoite-specific gene expression in the blood stages rather to the lack of alternative carbon sources in the malaria parasites, as suggested before (Chiappino-Pepe et al., 2017a). The genes that show essential liver phenotypes are related with fatty acid metabolism and TCA functions.

##### Identification of essential genes at alternative gene expression profiles for a scenario without regulation in iPbe

We integrated blood- and liver-stage gene expression data into iPbe using TEX-FBA and performed gene essentiality analyses at the expression profiles with maximum consistency score considering lack of regulation. We identified eight essential genes in addition to the blood- and liver-stage specific essential genes identified with gene expression data integrated when regulation of gene expression is allowed (Table S3, tab S3.2, column “Essential genes with gene expression data integrated when no regulation between isoenzymes is allowed (95-87)”). The essentiality of these eight genes is linked to the low expression level of their isoenzymes.

##### Identification of bottleneck reaction levels in iPbe

We looked for bottleneck reactions within the set of reactions associated with highly and lowly expressed genes based on the RNA-seq data and input parameters to TEX-FBA. We find a total of 107 and 159 *bottleneck reaction levels* or reaction levels in the blood and liver stages, respectively, that are responsible for the essential *in silico* function of the 64 and 70 essential genes when transcriptomics data are integrated into iPbe (Table S3).

##### From iPbe to iPbe-blood and iPbe-liver with PhenoMapping

We first defined a medium that includes all substrates present in the alternative IMMs (also called joint IMM) when the uptake of oxyhemoglobin is allowed (for the blood analysis) or not allowed (for the liver analysis). We performed essentiality analysis *in silico* at the joint IMM and the PhenoMapping analysis of bottleneck substrates. We identified the bottleneck substrates linked to dispensable phenotypes (based on the blood stage and liver stage data). These are substrates that should be added to the joint IMM to avoid false predictions of essentiality. We included such substrates in the joint IMM. We followed this approach to increase the consistency between *in silico* and *in vivo* observations and avoid increasing the disagreements. We additionally allowed the uptake of all amino acids and inorganics that were not part of the IMMs, since they are available in any growing condition. Based on this procedure, we suggest a medium of 90 and 94 allowed uptakes for the intraerythrocytic and intrahepatocytic trophozoite stages of *P. berghei*, respectively. We defined this media composition in iPbe-blood and iPbe-liver (see Table S4, tab 4.1, reactions defined as “drains / exchanges” in the “subSystem” column, and showing negative lower bound or *lb*).

We also defined the maximum uptake rate for 27 substrates that are carbon and purine sources in iPbe-blood and iPbe-liver (Table S4, uptakes with non-default lower bound or *lb*). Glucose is known to be the primary carbon source in the malaria parasite (Geary et al., 1985, Vander Jagt et al., 1990, Pfaller et al., 1982). The definition of uptake rates for the identified carbon sources is based on experimentally measured growth yields of *P. berghei* and *P. falciparum* on single carbon sources relative to the optimal growth yield on glucose (Geary et al., 1985). We limited the uptake of purine sources based on available kinetic data on the maximum uptake rate of some purines (Quashie et al., 2008). We assumed that the maximum uptake rate of adenosine is 10% of the maximum uptake rate of glucose. We defined the uptake rate of the remaining purine sources based on the relative value of the *V*_*max*_ uptake measured (Quashie et al., 2008). We also limited the uptake of the amino sugars based on an assumed uptake rate value relative to glucose to describe the slow phenotypes observed in the *Plasmo*GEM data. We calibrated the uptake of glucose and limited the uptake rates of the remaining carbon and purine sources relative to the glucose uptake rate to yield a specific growth of approximately 0.12 h^-1^ in iPbe-blood and 0.35 h^-1^ in iPbe-liver. We calculated the growth value assuming that 16 and 30,000 merozoites of *P. berghei* are formed in 24 and 30 h in the blood and liver stages, respectively.

We did not constrain the secretions in iPbe to generate iPbe-blood and iPbe-liver based on the following observation: out of the 43 genes essential in IMS conditions, 24 genes are also essential in IMM. From the 19 genes essential in the IMS that are not essential in any IMM, there is no gene with essential phenotype in the blood or liver stages. We hence allowed the models iPbe-blood and iPbe-liver to secrete all 248 extracellular metabolites.

We did not consider a lack of regulation of gene expression between isoenzymes in the final blood- and liver-stage specific models of iPbe based on the following observation: we reported eight new essential genes in a scenario without regulation of gene expression. Of those eight genes, only one (PBANKA_1109400) is essential in the blood stages and one (PBANKA_0820900) in the liver stages based on the available phenotypes.

We did not integrate metabolomics data since there was no dataset available for *P. berghei*.

To generate the final blood- and liver-stage specific iPbe models we relaxed the bottleneck reaction levels underlying dispensable phenotypes.

#### Pathway representation

The metabolic pathways depicted in this manuscript are based on their description in the genome-scale metabolic model of *P. berghei* iPbe (Table S4). Metabolites (medium size gray circles) are connected through metabolic reactions (solid arrows when single-step reactions are illustrated; else dashed arrows) and enzymes (white circles with enzyme abbreviated names) based on iPbe. We depicted the reaction directionalities obtained from a Flux Variablity Analysis (FVA) when 90% of optimal growth in the liver stages (iPbe-liver) is required. Extracellular metabolites (medium size circles with thick black lines) define which metabolites in the depicted pathway can be scavenged from or secreted to the surroundings/host cell in iPbe and transported into the *Plasmodium* intracellular compartment where the pathway is localized. Metabolites that are biomass precursors in iPbe-liver (black circles) describe the metabolic tasks associated with the present pathways in the liver stage development of the malaria parasites. All references to intracellular and extracellular metabolites refer to metabolites inside or outside the parasite cell. Blood ((Bushell et al., 2017), right box above the enzyme abbreviated name) and liver (this study, left box above the enzyme abbreviated name) phenotypes from the screening indicate whether the associated gene/s is/are dispensable (green), growth reducing (yellow), or essential (red) for *P. berghei* growth. A white box indicates that no phenotype is available for the corresponding gene and life stage. Enzyme abbreviated names as used in the Kyoto Encyclopedia of Genes and Genomes (KEGG (Kanehisa and Goto, 2000), http://www.genome.jp/kegg/) or PlasmoDB (Bahl et al., 2003).

### Quantification and Statistical Analysis

#### Analysis of barseq data

Comparison of data from the technical duplicate PCR reactions made from each gDNA sample allowed identification of reactions in which technical failures had occurred, meaning that PCRs were repeated. Sampling-induced error was modeled as follows: the log proportion of all barcodes in each sample was calculated as log_2_ (barcode count + 0.5), and the standard deviation between technical duplicates was calculated for each log-proportion. A moving average was taken by abundance with a window width of 11 across genes in the same sample. We enforced monotonicity on the relationship between abundance and standard deviation. For each stage transition, B1-M, M-SG, and SG-B2, we calculated a log_2_ fold change by subtracting the log-proportion in the former stage from the latter. We also propagated the errors associated with each of these measurements into an estimate of the error of this fold-change. In the case of the salivary gland to blood transition we additionally normalized for reductions that might be due to impaired blood stage growth, by correcting with a factor calculated from the Bushell et al., 2017 blood stage growth measurements of each gene, allowing for the three days of growth that mutants were given following sporozoite injection. Error values for blood stage growth measurements were again propagated.

Typically we now had three measurements for each transition for each gene. We amalgamated these into a single fold-change value by taking the inverse-variance weighted mean of the available values, and propagated errors through to this final value.

#### Variance analysis and phenotype assignment

We calculated proportions (relative abundance) for each gene in its respective pool for each sample, meaning the barcode count divided by the total barcode counts for that sample. We plotted relative abundance on a log2 scale in the MG sample against that in the B1 sample, coloring individual data points based on phenotype: no power, reduced, not reduced. We used violin plots to illustrate variance of relative abundances at the B1 stage and observed a higher variability and lower absolute relative abundance for the mutants with no power phenotype. This can be expected because if the absolute relative abundance is low at the B1 stage, this may lead to noisy measurements.

#### Statistical analysis of single gene KO phenotypic data

For oocyst number quantification, results were statistically evaluated using Prism (GraphPad) with a one-way analysis of variance (ANOVA) test with Dunnet’s multiple comparisons (^∗∗^p < 0.01, ^∗∗∗^p < 0.001, ^∗∗∗∗^p < 0.0001).

Relative sizes of liver stage parasites were statistically compared using Kruskal-Wallis tests; (^∗^p < 0.05, ^∗∗∗^p < 0.001).

Detached cell formation data were statistically evaluated using Prism (GraphPad) with a one-way analysis of variance (ANOVA) test with Dunnet’s multiple comparisons (^∗∗^p ≤ 0.01; ^∗∗∗^p ≤ 0.001).

### Data and Code Availability

Raw screening data as well as the downstream data processing workflow can be found in the following github repository: https://github.com/theosanderson/M-L_Screen.

The generic genome-scale metabolic model of *P. berghei* iPbe and the blood- and liver-stage specific models, i.e., iPbe-blood and iPbe-liver, in mat file format are available at the LCSB database http://lcsb-databases.epfl.ch/GEMs.

Documented implementation of the PhenoMapping workflow in MATLAB is available on www.github.com/EPFL-LCSB/phenomapping. The software package includes a tutorial that indicates step-by-step how to analyze context-specific information integrated into a genome-scale model using PhenoMapping. The genome-scale model iPbe and data used in this study are also available in the repository. The tutorials indicate how to reproduce the results of this paper. PhenoMapping requires the matTFA toolbox (Salvy et al., 2019) for TFA and TEX-FBA toolbox (Pandey et al., 2019) for the integration of gene expression data.

## Supplemental Information

Table S1. Relative Abundance (mean) and Standard for Genes Included in the Pilot Screen, Related to Figure 1

Table S2. M-L Screen Data, Related to Figure 2 Displayed is data pertaining to each gene analyzed in the M-L screen by barcoded knockout abundancy tracking. Data is shown for each stage transition; B1-MG, MG-SG, SG-B2. For each stage transition, non-normalized and normalized data is shown and for the SG-B2 transition, the following data is shown: non-normalized, normalized and normalized and then blood stage fitness-corrected. Within each stage transition, the Log2-fold-change (Log2-FC) is shown for each gene, the standard deviation of this value and whether this log2-FC is statistically significant in showing a reduction (reduced), whether there is no reduction (not reduced) or whether there is no statistical power associated with the log2-FC value. Where genes form part of the iPbe model, relevant metabolic subsystems are listed. GO Biological Process data was obtained from PlasmoDB (https://plasmodb.org/plasmo/).

Table S3. PhenoMapping Results for iPbe and Details on Its Reconstruction, Related to Figures 4, 5, 6, and 7 (1) Metabolic tasks for *P. berghei*; (2) Predictions of essentiality in iPbe (alternative conditions), iPbe-blood, and iPbe-liver; (3) *In silico* minimal media (IMM) results; (4) Essential genes in IMM and substrates responsible for essentiality; (5) *In silico* minimal secretion (IMS) results; (6) Essential genes in IMS and secretions responsible for essentiality; (7) Essential genes when metabolomics data are integrated and bottleneck metabolite levels; (8) Essential genes when transcriptomics data are integrated and bottleneck reaction levels; (9) List of false negative predictions with the final models iPbe, iPbe-blood, or iPbe-liver and hypotheses on the source for these mismatches; (10) Definition of biomass building blocks and stoichiometric requirements in iPbe; (11) Thermodynamic data for iPbe. All the PhenoMapping results can be reproduced following the PhenoMapping workflow available at https://www.github.com/EPFL-LCSB/phenomapping.

Table S4. Genome-Scale Metabolic Models iPbe, iPbe-Blood, and iPbe-Liver, Related to Figures 3, 4, 5, 6, and 7 (1) Metabolic reactions and associated information in iPbe, iPbe-blood, and iPbe-liver. We include enzyme abbreviations as presented in Figures 5, 6, and 7 for FASII, FAE and amino sugar pathways; (2) Metabolites participating in metabolic reactions in iPbe, iPbe-blood, and iPbe-liver; (3) Description of iPbe; (4) Measures for performance of iPbe-blood and iPbe-liver. The models (.mat files) for genome-wide analysis of *P. berghei* metabolism are available at the LCSB database https://lcsb-databases.epfl.ch/GEMs.

Table S5. Blood and Liver Sstage Screening Information Relating to Genes Involved in the FASII, FAE, and Amino Sugar Pathways, Related to Figures 5, 6, and 7

Table S6. Oligonucleotides Used for Genotyping of Single Knockout Mutants, Related to Figures 5, 6, and 7

Table S7. Index Primers Used for Multiplexing of Barseq Libraries, Related to Figures 5, 6, and 7
