# Supplementary material for: Genome-Scale Identification of Essential Metabolic Processes for Targeting the Plasmodium Liver Stage
Source: Cell. 2019 Nov 14;179(5):1112–1128.e26. doi: 10.1016/j.cell.2019.10.030 (PMC6904910; doi:10.1016/j.cell.2019.10.030)
Supplement: Table S1. Relative Abundance (mean) and Standard for Genes Included in the Pilot Screen, Related to Figure 1 [file mmc1.pdf]

Cell, Volume *179*

Supplemental Information

Genome-Scale Identification of Essential Metabolic Processes for Targeting the *Plasmodium* Liver Stage

Rebecca R. Stanway, Ellen Bushell, Anush Chiappino-Pepe, Magali Roques, Theo Sanderson, Blandine Franke-Fayard, Reto Caldelari, Murielle Golomingi, Mary Nyonda, Vikash Pandey, Frank Schwach, Séverine Chevalley, Jai Ramesar, Tom Metcalf, Colin Herd, Paul-Christian Burda, Julian C. Rayner, Dominique Soldati-Favre, Chris J. Janse, Vassily Hatzimanikatis, Oliver Billker, and Volker T. Heussler

Table S1: Relative abundance (mean) and standard for genes included in the pilot screen pilot screen, related to Figure 1. Displayed are the mean values (and standard deviations, SD) for relative of abundance of genes in the pilot screen. Those genes with a published liver stage phenotype are shown with an asterisk beside the Gene ID.

**Gene ID**	**Gene name**	**B1**		**MG**		**SG**		**B2**		**Reference related to liver stage phenotype**
		**Relative abundance**	**SD**	**Relative abundance**	**SD**	**Relative abundance**	**SD**	**Relative abundance**	**SD**	**-**
PBANKA_0514900	P28	1.000	0.201	0.900	0.266	0.926	0.193	2.197	0.565	-
PBANKA_0515000	P25	1.000	0.020	1.471	0.409	1.421	0.219	2.954	0.597	-
PBANKA_1034400	PM4	1.000	0.031	0.464	0.413	0.394	0.217	0.343	0.075	-
PBANKA_1037800	SOAP	1.000	0.385	1.232	0.128	1.332	0.293	2.725	0.654	-
PBANKA_1104200	BCKDHB	1.000	0.195	0.325	0.076	0.242	0.339	0.180	0.204	-
PBANKA_1401600	-	1.000	0.332	0.191	0.199	0.140	0.243	0.471	0.751	-
PBANKA_0315000	-	1.000	0.410	1.477	0.429	1.229	0.174	2.469	0.758	-
PBANKA_0401200	ABCB4	1.000	0.533	1.304	0.653	1.789	0.749	2.398	0.948	-
PBANKA_0519800	-	1.000	0.147	0.003	0.003	0.001	0.001	0.024	0.022	-
PBANKA_0833100	M18AAP	1.000	0.384	1.388	0.226	1.275	0.284	2.358	0.091	-
PBANKA_0933700	MAPK2	1.000	0.155	0.564	0.145	0.910	0.166	1.628	0.558	-
PBANKA_1038300	TRAM	1.000	0.197	0.965	0.226	0.772	0.123	1.038	0.100	-
PBANKA_1133400	EF-1alpha	1.000	0.417	1.117	0.092	1.072	0.135	2.667	1.717	-
PBANKA_1217700	DOZI	1.000	0.463	0.082	0.042	0.036	0.013	0.017	0.022	-
PBANKA_1357500	LipA	1.000	0.267	1.411	0.437	1.492	0.525	0.086	0.024	-
PBANKA_0615900	CRMP2	1.000	1.234	1.686	2.098	1.189	1.845	2.991	4.898	-
PBANKA_0702700	ROM3	1.000	0.146	2.491	0.509	0.669	0.086	1.401	0.262	-
PBANKA_0933500	ROM1	1.000	0.127	0.913	0.178	1.278	0.371	1.898	0.917	-

PBANKA_1411100	BCKDHA1	1.000	0.216	0.233	0.135	0.167	0.086	0.167	0.126	-
PBANKA_0101100*	PALM	1.000	0.282	1.259	0.157	1.289	0.023	0.081	0.029	(Haussig, *et al.*, 2011)
PBANKA_0501200*	UIS4	1.000	0.193	1.385	0.201	1.395	0.253	0.069	0.031	(Mueller et al., 2005)
PBANKA_0506500*	ZIPCO	1.000	0.143	0.716	0.212	0.584	0.150	0.282	0.040	(Sahu et al., 2014)
PBANKA_0714900*	aLipDH	1.000	0.410	1.708	0.206	1.526	0.304	0.075	0.027	(Pei et al., 2010)
PBANKA_0808100*	B9	1.000	0.103	1.612	0.612	1.581	0.288	0.086	0.043	(Annoura et al., 2014)
PBANKA_0902100*	SLARP	1.000	0.607	1.464	0.738	1.498	0.614	0.126	0.052	(Silvie, *et al.*, 2008)
PBANKA_1002100*	P36	1.000	0.234	1.286	0.322	1.526	0.184	0.074	0.034	(Ishino, *et al.*, 2005)
PBANKA_1002200*	P36p	1.000	0.234	1.076	0.378	1.552	0.392	0.060	0.024	(Ishino, *et al.*, 2005)
PBANKA_1006300*	PLP1	1.000	0.790	1.599	1.414	1.193	1.097	0.122	0.025	(Ishino, *et al.*, 2005a)
PBANKA_1024600*	LISP1	1.000	0.186	1.466	0.351	1.283	0.153	0.159	0.100	(Ishino et al., 2009)
PBANKA_1125100*	FabB/F	1.000	0.304	1.565	0.338	1.419	0.239	0.074	0.031	(Vaughan *et al.*, 2009)
PBANKA_1128100*	PL	1.000	0.363	1.221	0.299	1.346	0.476	0.636	0.559	(Burda et al., 2015)
PBANKA_1302800*	TPx1	1.000	0.241	1.400	0.115	1.299	0.125	2.169	0.494	(Yano et al., 2008)
PBANKA_1338200*	FabZ	1.000	0.059	1.515	0.303	1.425	0.289	0.079	0.035	(Vaughan *et al.*, 2009)
PBANKA_1349800*	TRAP	1.000	0.409	1.142	0.276	0.372	0.056	0.063	0.036	(Sultan et al., 1997)
