# Supplementary material for: Genome-Scale Identification of Essential Metabolic Processes for Targeting the Plasmodium Liver Stage
Source: Cell. 2019 Nov 14;179(5):1112–1128.e26. doi: 10.1016/j.cell.2019.10.030 (PMC6904910; doi:10.1016/j.cell.2019.10.030)
Supplement: Table S5. Blood and Liver Sstage Screening Information Relating to Genes Involved in the FASII, FAE, and Amino Sugar Pathways, Related to Figures 5, 6, and 7 [file mmc5.pdf]

Table S5: Blood and liver stage screening information relating to genes involved in the FASII, FAE, and amino sugar pathways, related to Figures 5, 6 and 7.

Participating genes	Enzyme name	Abbreviated name (pathway)	Screen phenotype statement		Liver stage screen information			
			Blood phenotype	Liver phenotype	Log2-fold change	Standard deviation	Statistical significance	Single knockout validation
**PBANKA_0308200**	apicoplast beta-ketoacyl-acyl carrier protein synthase III precursor, putative	**FabH (FASII)**	Slow	> 100-fold reduction	-9.61	1.02	reduced	Figures 5 and S4
**PBANKA_0923800**	pyruvate dehydrogenase E1 component subunit alpha	**PDH E1 alpha (FASII)**	Essential	no info				(Deschermeier et al., 2012)
**PBANKA_1310100**	pyruvate dehydrogenase E1 component subunit beta	**PDH E1 beta (FASII)**	no info	no info				
**PBANKA_0505000**	dihydrolipoamide acyltransferase, putative	**PDH E2 (FASII)**	Dispensable	> 100-fold reduction	-8.95	0.97	reduced	Figures 5 and S4
**PBANKA_0520000**	pyruvate kinase 2, putative	**PK (FASII)**	Essential	no info				
**PBANKA_0707000**	lipoate-protein ligase B	**LipB (FASII)**	Dispensable	> 100-fold reduction	-10.86	0.68	reduced	(Falkard et al., 2013)
**PBANKA_0823800**	3-oxoacyl-[acyl-carrier-protein] reductase, putative	**FabG (FASII)**	Dispensable	> 100-fold reduction	-9.23	0.99	reduced	Figures 5 and S4
**PBANKA_1125100**	3-oxoacyl-acyl-carrier protein synthase, putative	**FabB/F (FASII)**	Dispensable	2-100-fold reduction	-5.51	0.41	reduced	(Vaughan *et al.*, 2009), *P. yoelii*
**PBANKA_1229800**	enoyl-acyl carrier reductase	**FabI (FASII)**	no info	no info				(Yu et al., 2008)
**PBANKA_1332800**	biotin carboxylase subunit of acetyl CoA carboxylase, putative	**ACC (FASII)**	Essential	no info				
**PBANKA_1338200**	beta-hydroxyacyl-ACP dehydratase, putative	**FabZ (FASII)**	Dispensable	2-100-fold reduction	-4.68	0.23	reduced	(Vaughan *et al.*, 2009), *P. yoelii*
**PBANKA_1357500**	lipoyl synthase	**LipA (FASII)**	Dispensable	2-100-fold reduction	-5.09	0.35	reduced	Figures 5 and S4
**PBANKA_1410500**	malonyl CoA-acyl carrier protein transacylase precursor, putative	**FabD (FASII)**	Dispensable	> 100-fold reduction	-11.18	0.57	reduced	Figures 5 and S4
**PBANKA_0511000**	biotin--acetyl-CoA-carboxylase, putative	**HLCS (FASII)**	Dispensable	> 100-fold reduction	-8.17	0.63	reduced	(Dellibovi-Ragheb et al., 2018) and Fig. 5, Fig. S4
**PBANKA_0104700**	long chain polyunsaturated fatty acid elongation enzyme, putative	**ELO-B (FAE)**	Dispensable	not reduced	-0.57	1.02	no power	
**PBANKA_0204300**	fatty acid elongation protein, GNS1/SUR4 family, putative	**ELO-C (FAE)**	no info	no info				
**PBANKA_0522400**	steroid dehydrogenase, putative	**KCR (FAE)**	Dispensable*	> 100-fold reduction	-9.70	0.81	reduced*	Figures 6 and S5
**PBANKA_0820900**	long chain fatty acid elongation enzyme, putative	**ELO-A (FAE)**	Dispensable	> 100-fold reduction	-10.45	0.72	reduced	Figures 6 and S5

**PBANKA_0912700**	3-oxo-5-alpha-steroid 4-dehydrogenase, putative	**ECR (FAE)**	Essential	no info				
**PBANKA_1110700**	stearoyl-CoA desaturase, putative	**SCD (FAE)**	no info	no info				
**PBANKA_1143400**	NADH-cytochrome b5 reductase, putative	**CBR (FAE)**	Dispensable	> 100-fold reduction	-8.90	0.92	reduced	Figures 6 and S5
**PBANKA_1346500**	protein tyrosine phosphatase-like protein	**DEH (FAE)**	Dispensable	> 100-fold reduction	-10.34	0.65	reduced	(Guttery et al., 2014)
**PBANKA_0501700**	phosphomannomutase, putative	**PMM (AS)**	Dispensable	2-100-fold reduction	-2.58	0.19	reduced	Figures 7 and S6
**PBANKA_0509300**	glutamine--fructose-6-phosphate aminotransferase [isomerizing], putative	**GFPT (AS)**	Slow	not reduced	-1.41	1.32	no power	Mosquito stage arrest; not bypassed (Figure 7A)
**PBANKA_0918200**	phosphoacetylglucosamine mutase, putative	**PGM3 (AS)**	Slow	> 100-fold reduction	-7.77	0.57	reduced	Mosquito stage arrest; not bypassed (Figure 7A)
**PBANKA_1008800**	glucose-6-phosphate isomerase, putative	**GPI (AS)**	Essential	no info				
**PBANKA_1022300**	mannose-1-phosphate guanyltransferase, putative	**GMPP (AS)**	Slow	not reduced	3.93	0.45	not reduced	
**PBANKA_1122900**	hexokinase, putative	**HK (AS)**	Essential	no info				
**PBANKA_1127600**	N-acetyltransferase, GNAT family, putative	**GNPNAT / GNA1 (AS)**	no info	no info				
**PBANKA_1210900**	phosphoglucomutase, putative	**PGM (AS)**	Dispensable	2-100-fold reduction	-2.04	0.32	reduced	
**PBANKA_1212400**	GDP-L-fucose synthase, putative	**TSTA (AS)**	Dispensable	not reduced	0.08	0.18	not reduced	
**PBANKA_1228400**	mannose-6-phosphate isomerase, putative	**MPI (AS)**	Slow	not reduced	1.12	0.41	not reduced	
**PBANKA_1232300**	UTP--glucose-1-phosphate uridylyltransferase, putative	**USP (AS)**	Dispensable	not reduced	0.37	0.73	not reduced	Figures 7 and S6
**PBANKA_1343800**	transcriptional regulatory protein sir2a	**GNPDA (AS)**	Dispensable	not reduced	0.54	0.47	no power	
**PBANKA_1356600**	UDP-N-acetylglucosamine pyrophosphorylase, putative	**UAP (AS)**	Slow	> 100-fold reduction	-8.11	1.36	reduced	Figures 7 and S6
**PBANKA_1423700**	GDP-mannose 4,6-dehydratase, putative	**GMDS (AS)**	Dispensable	not reduced	-0.27	0.17	not reduced	
**PBANKA_0715500**	phosphoglucomutase-2	**PGM (AS)**	Slow	not reduced	0.08	0.67	no power	

Note on *: Having established that the ΔKCR knock-out mutant does not show a significant blood stage fitness defect (Figure S5A), the SG-B2 log2-fold change value shown here is that prior to blood stage fitness correction and thus not factoring in the “essential” blood stage phenotype determined by Bushell *et al*., 2017.
