# Supplementary material for: Genome-Scale Identification of Essential Metabolic Processes for Targeting the Plasmodium Liver Stage
Source: Cell. 2019 Nov 14;179(5):1112–1128.e26. doi: 10.1016/j.cell.2019.10.030 (PMC6904910; doi:10.1016/j.cell.2019.10.030)
Supplement: Table S6. Oligonucleotides Used for Genotyping of Single Knockout Mutants, Related to Figures 5, 6, and 7 [file mmc6.pdf]

Table S6: Oligonucleotides used for genotyping of single knock-out mutants, related to Figures 5, 6 and 7.

Gene ID	PbGEM vector	Gene name	Primer name	Sequence 5'→3'	Description	Size PCR product
**Genotyping for mutants generated with a 3xHA-hdhfr-yFCU gene replacement cassette**						
PBANKA_0505000	PbGEM-085541	PDH-E2	8947	ATGGTTCCCATGCTGCTACA	QCR1, Sanger	300 bp
			8948	AAACGCTTCGGGGCTATACT	QCR2, Sanger	
PBANKA_0511000	PbGEM-015012	HCS1	8360	GCACGGCACTTGCATTTCGATG	5'F, LUMC	733 bp
			8361	CATCATGTAATATGCCTTTTAAG	3'R, LUMC	
PBANKA_1410500	PbGEM-063795	FabD	8362	GACAAGTGTAATAATGATGTAG	5'F, LUMC	844 bp
			8363	CATTATAAGCACAACCATCTAC	3'R, LUMC	
PBANKA_0823800	PbGEM-342140	FabG	8370	CATTCCTTTATTTTAGGTTAATC	5'F, LUMC	972 bp
			8371	CATTCTTCCAGCAGGAATATTCG	3'R, LUMC	
PBANKA_0308200	PbGEM-270259	FabH	8366	CTAGAATACGCGCTGTTGGTGG	5'F, LUMC	656 bp
			8367	CTCCAAATAAATTTTGAGGCG	3'R, LUMC	
PBANKA_1357500	PbGEM-317331	LipA	8368	GCATATTTGCTCTGCCTTATTC	5'F, LUMC	918 bp
			8369	GGGCATATACATCTAACCCAC	3'R, LUMC	
PBANKA_0820900	PbGEM-239778	ELO-A	8364	GGATCCCAGGAAAGGGGAGAG	5'F, LUMC	1 kb
			8365	GTCATGGGCATATGTTGCAAATC	3'R, LUMC	
PBANKA_0522400	PbGEM-538163	KCR	8576	AGCTCGGTTTCCATTTCGTG	QCR1, Sanger	484 bp
			8577	ACAGGATGCACGGATGGTATTGGG	QCR2, Sanger	
PBANKA_1143400	PbGEM-342764	CBR	8951	TCCACTTACCTTTCCCACAAACA	QCR1, Sanger	523 bp
			8952	CCGACCAGCTGTATTATACATGTGC	QCR2, Sanger	
PBANKA_1232300	PbGEM-313053	USP	8745	TGGAACCCCATATATTATGCCACCCA	QCR1, Sanger	611 bp
			8746	TGAAATGAACGCAGGGATGT	QCR2, Sanger	
PBANKA_0501700	PbGEM-341900	PMM	8735	AGGTCCCATCTACATCGAACAGA	QCR1, Sanger	638 bp
			8736	AAGTGCATTTGGTCAATCCA	QCR2, Sanger	
PBANKA_0918200	PbGEM-093581	PGM3	8739	AGCTCGAAAATCTTCCATAGGT	QCR1, Sanger	518 bp
			8740	AGTCAACTACAGCTTTTCGAACGT	QCR2, Sanger	
PBANKA_0509300	PbGEM-274634	GFPT	9168	CAGAGGATATGATTCATGTGGTATG	5'F, LUMC	803 bp
			9169	CATCGACGTATGGGTCTAAACCACC	3'R, LUMC	
**Genotyping for mutants generated with a GOMO-GFP-Cherry-FACS gene replacement cassette**						
PBANKA_0501700	PbGEM-646744	PMM	1593	TCCAAAAGAATGTGCCAACTCGT	GT Sanger Integration PCR with 1431	6.5 kb
			1591	AGGTCCCATCTACATCGAA	QCR1 Sanger	700 bp
			1592	AAGTGCATTTGGTCAATCCA	QCR2 Sanger	
PBANKA_1356600	PbGEM-646750	UAP	1596	TGTTGTTCCCATCCTCTGTTG	GT Sanger Integration PCR with 1752	5 kb
			1594	TGAAGTCGTGATCCCAACCC	QCR1 Sanger	500 bp
			1595	CAGGCGTTCATCATTGACCTAC	QCR2 Sanger	
PBANKA_0522400	PbGEM-645640	KCR	1578	ACATGTGCATGTGTATGTGCT	GT Sanger Integration PCR with 1431	6 kb
			1577	AGCTCGGTTTCCATTTCGTG	QCR1 Sanger	200 bp
			1576	ACAGGATGCACGGATGGTATTG	QCR2 Sanger	
**Integration primers used with GT**						
-	-	3'*Pb*DHFR	1752	CACACATAAAATGGCTAGTATGAATAGC	GW1	
		3'*Pb*Hsp70	1431	CTTTGGTGACAGATACTACTGTGTTT	GW2	
**Pb gDNA control primers**						
PBANKA_0514900	-	P28	1755	CGGCCATGGATGAATTTTAAATACA		200 bp
			1271	TCCCCGCGGCCATGGATGAATTTTAAATACAGTTTTATT		
			1756	GGACTAGTTGGCTTACATATATTTTGTG		
