# Supplementary material for: Genome-Scale Identification of Essential Metabolic Processes for Targeting the Plasmodium Liver Stage
Source: Cell. 2019 Nov 14;179(5):1112–1128.e26. doi: 10.1016/j.cell.2019.10.030 (PMC6904910; doi:10.1016/j.cell.2019.10.030)
Supplement: Table S7. Index Primers Used for Multiplexing of Barseq Libraries, Related to Figures 5, 6, and 7 [file mmc7.pdf]

Table S7: Index primers used for multiplexing of barseq libraries, related to Figures 5, 6 and 7.

**Oligo name**	**Sequence 5'→3'**	**Barcode**
Arg00444	TCGGCATTCCTGCTGAACCGCTCTTCCGATCTGTAATTCGTGCGCGTCAG	-
Arg00445	ACACTCTTTCCCTACACGACGCTCTTCCGATCTCCTTCAATTTCGATGGGTAC	-
PE 1.0	AATGATACGGCGACCACCGAGATCTACACTCTTTCCCTACACGACGCTCTTCCGATC*T	-
iPCRindex1	CAAGCAGAAGACGGCATACGAGATTGCTAATCACTGAGATCGGTCTCGGCATTCCTGCTGAACCGCTCTTCCGATC*T	AGTGATTAGCA
iPCRindex2	CAAGCAGAAGACGGCATACGAGATTAGGGGGATTCGAGATCGGTCTCGGCATTCCTGCTGAACCGCTCTTCCGATC*T	GAATCCCCCTA
iPCRindex3	CAAGCAGAAGACGGCATACGAGATAGTTTCCCAGGGAGATCGGTCTCGGCATTCCTGCTGAACCGCTCTTCCGATC*T	CCTGGGAAACT
iPCRindex4	CAAGCAGAAGACGGCATACGAGATCCTGGGAGGTAGAGATCGGTCTCGGCATTCCTGCTGAACCGCTCTTCCGATC*T	TACCTCCCAGG
iPCRindex5	CAAGCAGAAGACGGCATACGAGATATACCACAAATGAGATCGGTCTCGGCATTCCTGCTGAACCGCTCTTCCGATC*T	ATTTGTGGTAT
iPCRindex6	CAAGCAGAAGACGGCATACGAGATGATCTCTCGGGGAGATCGGTCTCGGCATTCCTGCTGAACCGCTCTTCCGATC*T	CCCGAGAGATC
iPCRindex7	CAAGCAGAAGACGGCATACGAGATACCCTATACTCGAGATCGGTCTCGGCATTCCTGCTGAACCGCTCTTCCGATC*T	GAGTATAGGGT
iPCRindex8	CAAGCAGAAGACGGCATACGAGATCTCAATTAAGAGAGATCGGTCTCGGCATTCCTGCTGAACCGCTCTTCCGATC*T	TCTTAATTGAG
iPCRindex9	CAAGCAGAAGACGGCATACGAGATCGACAGAACGTGAGATCGGTCTCGGCATTCCTGCTGAACCGCTCTTCCGATC*T	ACGTTCTGTCG
iPCRindex10	CAAGCAGAAGACGGCATACGAGATTCGCCATTATGGAGATCGGTCTCGGCATTCCTGCTGAACCGCTCTTCCGATC*T	CATAATGGCGA
iPCRindex11	CAAGCAGAAGACGGCATACGAGATATGTTCCGGCCGAGATCGGTCTCGGCATTCCTGCTGAACCGCTCTTCCGATC*T	GGCCGGAACAT
iPCRindex12	CAAGCAGAAGACGGCATACGAGATTCTTGAAGTGAGAGATCGGTCTCGGCATTCCTGCTGAACCGCTCTTCCGATC*T	TCACTTCAAGA
iPCRindex13	CAAGCAGAAGACGGCATACGAGATGAAGGCCAGCTGAGATCGGTCTCGGCATTCCTGCTGAACCGCTCTTCCGATC*T	AGCTGGCCTTC
iPCRindex14	CAAGCAGAAGACGGCATACGAGATCCAATGTGCAGGAGATCGGTCTCGGCATTCCTGCTGAACCGCTCTTCCGATC*T	CTGCACATTGG
iPCRindex15	CAAGCAGAAGACGGCATACGAGATATCGAAGGACCGAGATCGGTCTCGGCATTCCTGCTGAACCGCTCTTCCGATC*T	GGTCCTTCGAT
iPCRindex16	CAAGCAGAAGACGGCATACGAGATTCGGGTGCGAAGAGATCGGTCTCGGCATTCCTGCTGAACCGCTCTTCCGATC*T	TTCGCACCCGA
iPCRindex17	CAAGCAGAAGACGGCATACGAGATGTAATTTACGGGAGATCGGTCTCGGCATTCCTGCTGAACCGCTCTTCCGATC*T	CCGTAAATTAC
iPCRindex18	CAAGCAGAAGACGGCATACGAGATATATCGACTACGAGATCGGTCTCGGCATTCCTGCTGAACCGCTCTTCCGATC*T	GTAGTCGATAT
iPCRindex19	CAAGCAGAAGACGGCATACGAGATTGATTCTTACAGAGATCGGTCTCGGCATTCCTGCTGAACCGCTCTTCCGATC*T	TGTAAGAATCA
iPCRindex20	CAAGCAGAAGACGGCATACGAGATACGGCGGGCCTGAGATCGGTCTCGGCATTCCTGCTGAACCGCTCTTCCGATC*T	AGGCCCGCCGT
iPCRindex21	CAAGCAGAAGACGGCATACGAGATCTTGCGTGGAGGAGATCGGTCTCGGCATTCCTGCTGAACCGCTCTTCCGATC*T	CTCCACGCAAG
iPCRindex22	CAAGCAGAAGACGGCATACGAGATTAATCAAAGACGAGATCGGTCTCGGCATTCCTGCTGAACCGCTCTTCCGATC*T	GTCTTTGATTA
iPCRindex23	CAAGCAGAAGACGGCATACGAGATGGCGGGCTCTAGAGATCGGTCTCGGCATTCCTGCTGAACCGCTCTTCCGATC*T	TAGAGCCCGCC
iPCRindex24	CAAGCAGAAGACGGCATACGAGATCCTCCATTTCTGAGATCGGTCTCGGCATTCCTGCTGAACCGCTCTTCCGATC*T	AGAAATGGAGG
iPCRindex25	CAAGCAGAAGACGGCATACGAGATAACCAGCGCTGGAGATCGGTCTCGGCATTCCTGCTGAACCGCTCTTCCGATC*T	CAGCGCTGGTT
iPCRindex26	CAAGCAGAAGACGGCATACGAGATTATTCGTCAACGAGATCGGTCTCGGCATTCCTGCTGAACCGCTCTTCCGATC*T	GTTGACGAATA
iPCRindex27	CAAGCAGAAGACGGCATACGAGATGCGCTGATGCAGAGATCGGTCTCGGCATTCCTGCTGAACCGCTCTTCCGATC*T	TGCATCAGCGC
iPCRindex28	CAAGCAGAAGACGGCATACGAGATCTCATATGGCTGAGATCGGTCTCGGCATTCCTGCTGAACCGCTCTTCCGATC*T	AGCCATATGAG
iPCRindex29	CAAGCAGAAGACGGCATACGAGATACAGGGGCAGGGAGATCGGTCTCGGCATTCCTGCTGAACCGCTCTTCCGATC*T	CCTGCCCCTGT
iPCRindex30	CAAGCAGAAGACGGCATACGAGATGGTTTTATACCGAGATCGGTCTCGGCATTCCTGCTGAACCGCTCTTCCGATC*T	GGTATAAAACC
iPCRindex31	CAAGCAGAAGACGGCATACGAGATGCATGACTTTAGAGATCGGTCTCGGCATTCCTGCTGAACCGCTCTTCCGATC*T	TAAAGTCATGC
iPCRindex32	CAAGCAGAAGACGGCATACGAGATTTCTGAGTTCTGAGATCGGTCTCGGCATTCCTGCTGAACCGCTCTTCCGATC*T	AGAACTCAGAA
iPCRindex33	CAAGCAGAAGACGGCATACGAGATCGATTAAGCTGGAGATCGGTCTCGGCATTCCTGCTGAACCGCTCTTCCGATC*T	CAGCTTAATCG
iPCRindex34	CAAGCAGAAGACGGCATACGAGATTCTCTTAAGCCGAGATCGGTCTCGGCATTCCTGCTGAACCGCTCTTCCGATC*T	GGCTTAAGAGA

iPCRindex35	CAAGCAGAAGACGGCATACGAGATCCGACAGGTGAGAGATCGGTCTCGGCATTCCTGCTGAACCGCTCTTCCGATC*T	TCACCTGTCGG
iPCRindex36	CAAGCAGAAGACGGCATACGAGATAGTATCACTATGAGATCGGTCTCGGCATTCCTGCTGAACCGCTCTTCCGATC*T	ATAGTGATACT
iPCRindex37	CAAGCAGAAGACGGCATACGAGATGTTCGCTGATGGAGATCGGTCTCGGCATTCCTGCTGAACCGCTCTTCCGATC*T	CATCAGCGAAC
iPCRindex38	CAAGCAGAAGACGGCATACGAGATTCCCATGGCGCGAGATCGGTCTCGGCATTCCTGCTGAACCGCTCTTCCGATC*T	GCGCCATGGGA
iPCRindex39	CAAGCAGAAGACGGCATACGAGATGGAGTTCAACAGAGATCGGTCTCGGCATTCCTGCTGAACCGCTCTTCCGATC*T	TGTTGAACTCC
iPCRindex40	CAAGCAGAAGACGGCATACGAGATACTGCGTATATGAGATCGGTCTCGGCATTCCTGCTGAACCGCTCTTCCGATC*T	ATATACGCAGT
iPCRindex41	CAAGCAGAAGACGGCATACGAGATTACGTCGTGCGGAGATCGGTCTCGGCATTCCTGCTGAACCGCTCTTCCGATC*T	CGCACGACGTA
iPCRindex42	CAAGCAGAAGACGGCATACGAGATCCTTCTGTCCCGAGATCGGTCTCGGCATTCCTGCTGAACCGCTCTTCCGATC*T	GGGACAGAAGG
iPCRindex43	CAAGCAGAAGACGGCATACGAGATATACTTGTTAAGAGATCGGTCTCGGCATTCCTGCTGAACCGCTCTTCCGATC*T	TTAACAAGTAT
iPCRindex44	CAAGCAGAAGACGGCATACGAGATTACCCAGGAGTGAGATCGGTCTCGGCATTCCTGCTGAACCGCTCTTCCGATC*T	ACTCCTGGGTA
iPCRindex45	CAAGCAGAAGACGGCATACGAGATGGGGTTTTCTGGAGATCGGTCTCGGCATTCCTGCTGAACCGCTCTTCCGATC*T	CAGAAAACCCC
iPCRindex46	CAAGCAGAAGACGGCATACGAGATACTGCTCGTGCGAGATCGGTCTCGGCATTCCTGCTGAACCGCTCTTCCGATC*T	GCACGAGCAGT
iPCRindex47	CAAGCAGAAGACGGCATACGAGATGAAGCATAATAGAGATCGGTCTCGGCATTCCTGCTGAACCGCTCTTCCGATC*T	TATTATGCTTC
iPCRindex48	CAAGCAGAAGACGGCATACGAGATATACAGTCGCTGAGATCGGTCTCGGCATTCCTGCTGAACCGCTCTTCCGATC*T	AGCGACTGTAT
iPCRindex49	CAAGCAGAAGACGGCATACGAGATCTGTCGCAAGGGAGATCGGTCTCGGCATTCCTGCTGAACCGCTCTTCCGATC*T	CCTTGCGACAG
iPCRindex50	CAAGCAGAAGACGGCATACGAGATTGCAATCTAACGAGATCGGTCTCGGCATTCCTGCTGAACCGCTCTTCCGATC*T	GTTAGATTGCA
iPCRindex51	CAAGCAGAAGACGGCATACGAGATGATGGTATTTAGAGATCGGTCTCGGCATTCCTGCTGAACCGCTCTTCCGATC*T	TAAATACCATC
iPCRindex52	CAAGCAGAAGACGGCATACGAGATCTAAAAAATATGAGATCGGTCTCGGCATTCCTGCTGAACCGCTCTTCCGATC*T	ATATTTTTTAG
iPCRindex53	CAAGCAGAAGACGGCATACGAGATAGCGTTTAGGGGAGATCGGTCTCGGCATTCCTGCTGAACCGCTCTTCCGATC*T	CCCTAAACGCT
iPCRindex54	CAAGCAGAAGACGGCATACGAGATTCCTAACAATCGAGATCGGTCTCGGCATTCCTGCTGAACCGCTCTTCCGATC*T	GATTGTTAGGA
iPCRindex55	CAAGCAGAAGACGGCATACGAGATGAGACTCGGTAGAGATCGGTCTCGGCATTCCTGCTGAACCGCTCTTCCGATC*T	TACCGAGTCTC
iPCRindex56	CAAGCAGAAGACGGCATACGAGATCAATTTGTTCTGAGATCGGTCTCGGCATTCCTGCTGAACCGCTCTTCCGATC*T	AGAACAAATTG
iPCRindex57	CAAGCAGAAGACGGCATACGAGATATGAGATCAAGGAGATCGGTCTCGGCATTCCTGCTGAACCGCTCTTCCGATC*T	CTTGATCTCAT
iPCRindex58	CAAGCAGAAGACGGCATACGAGATTAACGCGTGGCGAGATCGGTCTCGGCATTCCTGCTGAACCGCTCTTCCGATC*T	GCCACGCGTTA
iPCRindex59	CAAGCAGAAGACGGCATACGAGATCCGTATCTATAGAGATCGGTCTCGGCATTCCTGCTGAACCGCTCTTCCGATC*T	TATAGATACGG
iPCRindex60	CAAGCAGAAGACGGCATACGAGATTGGCTGGGTATGAGATCGGTCTCGGCATTCCTGCTGAACCGCTCTTCCGATC*T	ATACCCAGCCA
iPCRindex61	CAAGCAGAAGACGGCATACGAGATGTACATCCGGGGAGATCGGTCTCGGCATTCCTGCTGAACCGCTCTTCCGATC*T	CCCGGATGTAC
iPCRindex62	CAAGCAGAAGACGGCATACGAGATAAGCCGAGATCGAGATCGGTCTCGGCATTCCTGCTGAACCGCTCTTCCGATC*T	GATCTCGGCTT
iPCRindex63	CAAGCAGAAGACGGCATACGAGATCAAAGCGGCTAGAGATCGGTCTCGGCATTCCTGCTGAACCGCTCTTCCGATC*T	TAGCCGCTTTG
iPCRindex64	CAAGCAGAAGACGGCATACGAGATGCTGTATTTAGGAGATCGGTCTCGGCATTCCTGCTGAACCGCTCTTCCGATC*T	CTAAATACAGC
